# FUT2-mediated intestinal fucosylation: a master regulator of host-microbiota symbiosis in health and disease

**DOI:** 10.3389/fmicb.2026.1926158

**Published:** 2026-08-20

**Authors:** Lifen Zhao, Nianzhu Zhang

**Affiliations:** Department of Laboratory Medicine, The Second Hospital of Dalian Medical University, Dalian, Liaoning, China

**Keywords:** fucosyltransferase 2, gut microbiota, human milk oligosaccharides, intestinal fucosylation, secretor status

## Abstract

Intestinal fucosylation, the enzymatic addition of fucose to glycoconjugates, represents a pivotal regulatory mechanism that modulates host–microbiota crosstalk in the gastrointestinal tract. This review comprehensively synthesizes current advances in the molecular mechanisms, multilevel regulatory networks, and functional implications of intestinal mucosal fucosylation. This review focuses on α(1,2)-fucosylation catalyzed by fucosyltransferase 2 (FUT2)—a reaction that determines the secretor status and modifies critical glycoconjugates, including mucins and human milk oligosaccharides. This review examines the dynamic regulation of epithelial fucosylation by host-derived factors—including immune mediators [notably interleukin-22 (IL-22) secreted by group 3 innate lymphoid cells (ILC3s)], the enteric nervous system (ENS), and microbial signals. Furthermore, this review details how fucosylated glycans shape the gut microbial ecosystem by serving as selective nutrient sources for beneficial symbionts—including *Bifidobacterium* and *Bacteroides* species—while concurrently enhancing colonization resistance against enteric pathogens. Dysregulation of this axis is increasingly associated with a spectrum of human pathologies, including inflammatory bowel disease (IBD), metabolic dysfunction, and enteric infections. Finally, this review discusses emerging therapeutic strategies targeting the modulation of intestinal fucosylation and its downstream physiological consequences, with particular emphasis on personalized approaches informed by host genetic determinants—most notably *FUT2* secretor status.

## 1 Introduction: host-microbe symbiosis

Glycosylation mediates the formation of the host’s “glycocalyx” (Crouch et al., 2024), a structurally and functionally specialized glycan layer situated at the mucosal interface, which plays a pivotal role in modulating host–microorganism interactions (Crouch et al., 2024; Demirturk et al., 2024). The deoxyhexose L-fucose holds particular physiological significance in the gastrointestinal tract (Garber et al., 2021). Fucosylation—the enzymatic addition of L-fucose residues—frequently targets terminal positions of glycan chains, including mucin-type O-glycans, thereby rendering these glycans accessible to luminal microbes (Rahfeld et al., 2019).

Intestinal fucosylation is mediated not by a single enzyme but by several fucosyltransferases (FUTs) with distinct acceptor specificities: FUT1 and FUT2 catalyze terminal α1,2-fucosylation—FUT1 contributing baseline fucosylation on Paneth cells and Peyer’s patch M cells (Terahara et al., 2011; Kamioka et al., 2022); FUT3 (the Lewis enzyme) catalyzes α1,3/α1,4-fucosylation to generate Lewis antigens (Orczyk-Pawiłowicz and Lis-Kuberka, 2020), whereas FUT8 catalyzes α1,6-linked core fucosylation of N-glycans, a modification required for T-cell receptor signaling (Fujii et al., 2016). The *FUT2* gene encodes α(1,2)-fucosyltransferase, the key enzyme responsible for catalyzing α(1,2)-fucosylation in human gastrointestinal mucosa and secreted glycoconjugates (Hu et al., 2022). Whereas homozygous loss-of-function variants in *FUT2* result in the non-secretor phenotype, individuals retaining at least one functional allele exhibit the secretor phenotype, characterized by expression of α(1,2)-fucosylated glycan structures including the H antigen (Rausch et al., 2011; Hu et al., 2022). This genetic variation establishes functionally distinct ecological niches within the gastrointestinal habitat, thereby exerting a selective influence on microbiota composition and structure (Crouch et al., 2024; Wang et al., 2024). Intestinal fucosylation is dynamic, with specialized cells like FUT2-expressing (FUT2^+^) Paneth cells generating cohabitation niches for commensals (Kamioka et al., 2022).

Fucosylated glycans present on secreted mucins—particularly mucin 2 (MUC2)—and human milk oligosaccharides (HMOs) function dually as nutrient substrates and molecular docking sites for commensal and symbiotic microbes (Garber et al., 2021; Fancy et al., 2024). Fucosylated HMOs derived from secretor-status mothers function as selective prebiotic substrates that enrich beneficial, fucose-utilizing commensal bacteria—such as *Bifidobacterium longum* subsp. *infantis*—in the developing infant gut microbiota (Xu et al., 2025). Specialized commensals, like certain *Bifidobacterium* species, use specific transporters to utilize these substrates, giving them a competitive advantage (Sakanaka et al., 2019). For instance, *Bifidobacterium infantis* colonization is strongly associated with the host’s *Fut2* secretor status (Wang et al., 2024).

Unless otherwise specified, “fucosylation” throughout this manuscript refers exclusively to host-mediated post-translational glycosylation during protein maturation, catalyzed by fucosyltransferases (FUTs) in the Golgi apparatus and covalently linked to mucins, glycolipids, or other glycoconjugates prior to their secretion or surface presentation. We do not discuss secondary modifications of pre-formed glycans occurring *de novo* within the intestinal lumen (e.g., microbial fucosidase-mediated trimming or salvage pathway re-incorporation of free fucose into nascent glycans), except where explicitly noted in the context of microbial metabolism. Furthermore, the term “terminal fucosylation” denotes the presence of fucose as a non-reducing terminal residue on glycan chains, rather than implying apical or lateral cellular localization. Fucosylation fulfills a dual functional role: it serves as a nutrient source for beneficial symbionts, yet simultaneously provides exploitable glycan epitopes that facilitate the adhesion, colonization, and virulence of certain enteric pathogens. For example, *Campylobacter jejuni* (*C. jejuni*) binds the α(1,2)-fucosylated H-2 antigen to initiate infection (Ruiz-Palacios et al., 2003). Conversely, soluble fucosylated human milk oligosaccharides (fHMOs) function as molecular decoys that competitively inhibit pathogen adhesion to host epithelial glycans, thereby conferring protection against enteric infections—including gastroenteritis—in breastfed infants (Ruiz-Palacios et al., 2003; Rubinstein et al., 2025). Fucosylation is regulated by host genetics and by immune and nervous system signals. Group 3 innate lymphoid cells (ILC3s) induce epithelial *Fut2* expression via interleukin-22 (IL-22), a mechanism critical for resistance against *Salmonella Typhimurium* (Goto et al., 2014). Moreover, vasoactive intestinal peptide (VIP)–producing enteric neurons directly stimulate epithelial *Fut2* expression via neuronal–epithelial crosstalk (Lei et al., 2022). Notably, intestinal glycans undergo simultaneous modifications including sialylation, sulfation, and core N-glycan fucosylation, which often mask or synergize with α1,2-fucosylation effects. Current studies rarely dissect the independent contribution of fucosylation from these co-occurring modifications, particularly in mucosal niches where glycan structures are spatially heterogeneous—a key limitation for causal inference of fucosylation-specific effects in disease. Although glycosylation broadly supports glycocalyx formation across diverse tissues—most notably the proteoglycan-rich endothelial glycocalyx regulating vascular permeability and leukocyte trafficking—this review is intentionally restricted to the intestinal mucosal glycocalyx. This specialized structure is defined by its dense, microbe-facing matrix of gel-forming mucins (e.g., MUC2), where fucosylation plays a dominant role in mediating host-microbiota interactions rather than hemodynamic regulation. Within this broader enzymatic landscape, this review focuses on FUT2-mediated α1,2-fucosylation, a choice motivated by the unique responsiveness of FUT2 to environmental cues: the gut microbiota selectively induces *Fut2* expression while leaving other fucosyltransferases—including *Fut1* and *Fut8*—unaffected (Tsang et al., 2022), and epithelial *Fut2* is further upregulated by ILC3-derived IL-22 and VIP-producing enteric neurons (Goto et al., 2014; Lei et al., 2022). FUT2 thus constitutes the principal dynamically regulated axis of intestinal fucosylation; throughout this review, FUT2-dependent mechanisms are distinguished from intestinal fucosylation more broadly. This review comprehensively examines the multifaceted roles of intestinal fucosylation in shaping host-associated microbial ecology, mediating mutualistic symbiosis, and modulating susceptibility to infectious and inflammatory diseases.

## 2 The molecular machinery and multi-level regulation of intestinal fucosylation

### 2.1 Fucosyltransferases (FUTs)

*De novo* GDP-fucose synthesis begins with the conversion of fructose-6-phosphate to mannose-1-phosphate via the hexosamine pathway, followed by sequential actions of GDP-mannose 4,6-dehydratase (GMDS) and fucose synthase (FX). An alternative salvage pathway recycles free L-fucose from dietary sources or glycan turnover via fucose kinase (FK) and GDP-fucose pyrophosphorylase (GFPP), generating the universal donor substrate GDP-fucose for all FUTs. Fucosylation is catalyzed by a family of FUTs, which transfer fucose from GDP-fucose to specific acceptor glycans in a linkage- and site-specific manner (Table 1). As defined in the Introduction, this enzymatic activity occurs during host protein maturation in the secretory pathway, distinct from post-secretory modifications in the intestinal lumen. *De novo* synthesis of GDP-fucose—catalyzed by GMDS and FX—is indispensable for intestinal fucosylation; mice with genetic ablation of *FX* exhibit complete loss of epithelial fucosylation, spontaneous colitis, and progressive adenocarcinoma, phenotypes that are fully rescued by dietary fucose supplementation (Wang et al., 2017). The gut microbiota induces the expression of FUTs, but not the GDP-fucose synthesis genes *Gmds* or *Fx* (Tsang et al., 2022). Microbial signals also regulate additional components of the intestinal glycosylation machinery, including the molecular chaperone C1GALT1C1 (Cosmc), which is essential for the proper folding and stability of core 1 β3-galactosyltransferase (C1GALT1) during O-glycan biosynthesis (Yao et al., 2025). These pathways compete for shared upstream precursors: UDP-GlcNAc serves as a common substrate for both GDP-fucose and CMP-sialic acid synthesis, so upregulated fucosylation restricts sialylation by limiting precursor availability, and vice versa.

**TABLE 1:** Key molecules in the regulation and machinery of intestinal fucosylation.

Name	Content/function	Citation (PMID)
GMDS / FX	Enzymes for *de novo* synthesis of guanosine diphosphate–fucose (GDP-fucose). *FX* deficiency leads to colitis and adenocarcinoma.	(Wang et al., 2017)
C1GALT1C1 (Cosmc)	O-glycan synthesis chaperone regulated by microbial signals.	(Yao et al., 2025)
FUT2	α1,2-fucosyltransferase; creates H-type 1 antigen (secretor status). Induced by microbiota/IL-22 on epithelial and specialized Paneth cells.	(Goto et al., 2014; Kamioka et al., 2022; Lei et al., 2022; Tsang et al., 2022)
FUT1	α1,2-fucosyltransferase; provides baseline fucosylation on Paneth cells and fucosylates Peyer’s patch M cells.	(Terahara et al., 2011; Kamioka et al., 2022)
FUT8	α1,6-fucosyltransferase (core fucosylation); required for T-cell receptor signaling. Deficiency protects from T-cell-mediated colitis.	(Fujii et al., 2016)
ILC3s	Innate lymphoid cells that induce epithelial *Fut2* expression via IL-22 and lymphotoxin.	(Goto et al., 2014)
IL-22	Cytokine produced by ILC3s; drives *Fut2* expression, development of Fut2+ Paneth cells, and colonization resistance.	(Goto et al., 2014; Pham et al., 2014; Pickard et al., 2014; Kamioka et al., 2022)
IL-23	Cytokine produced by dendritic cells; activates ILC3s to secrete IL-22 during systemic stress.	(Pickard et al., 2014)
Lymphotoxin	Mediates microbiota-independent induction of *Fut2* by ILC3s.	(Goto et al., 2014)

GMDS, GDP-mannose-4,6-dehydratase; FX, GDP-4-keto-6-deoxymannose 3,5-epimerase/4-reductase (GDP-L-fucose synthase); C1GALT1C1, core 1 β1,3-galactosyltransferase-specific chaperone (Cosmc); FUT, fucosyltransferase; ILC3, group 3 innate lymphoid cell; IL, interleukin; PMID, PubMed identifier. α1,2- and α1,6- denote fucose glycosidic linkage positions.

**FIGURE 1:**
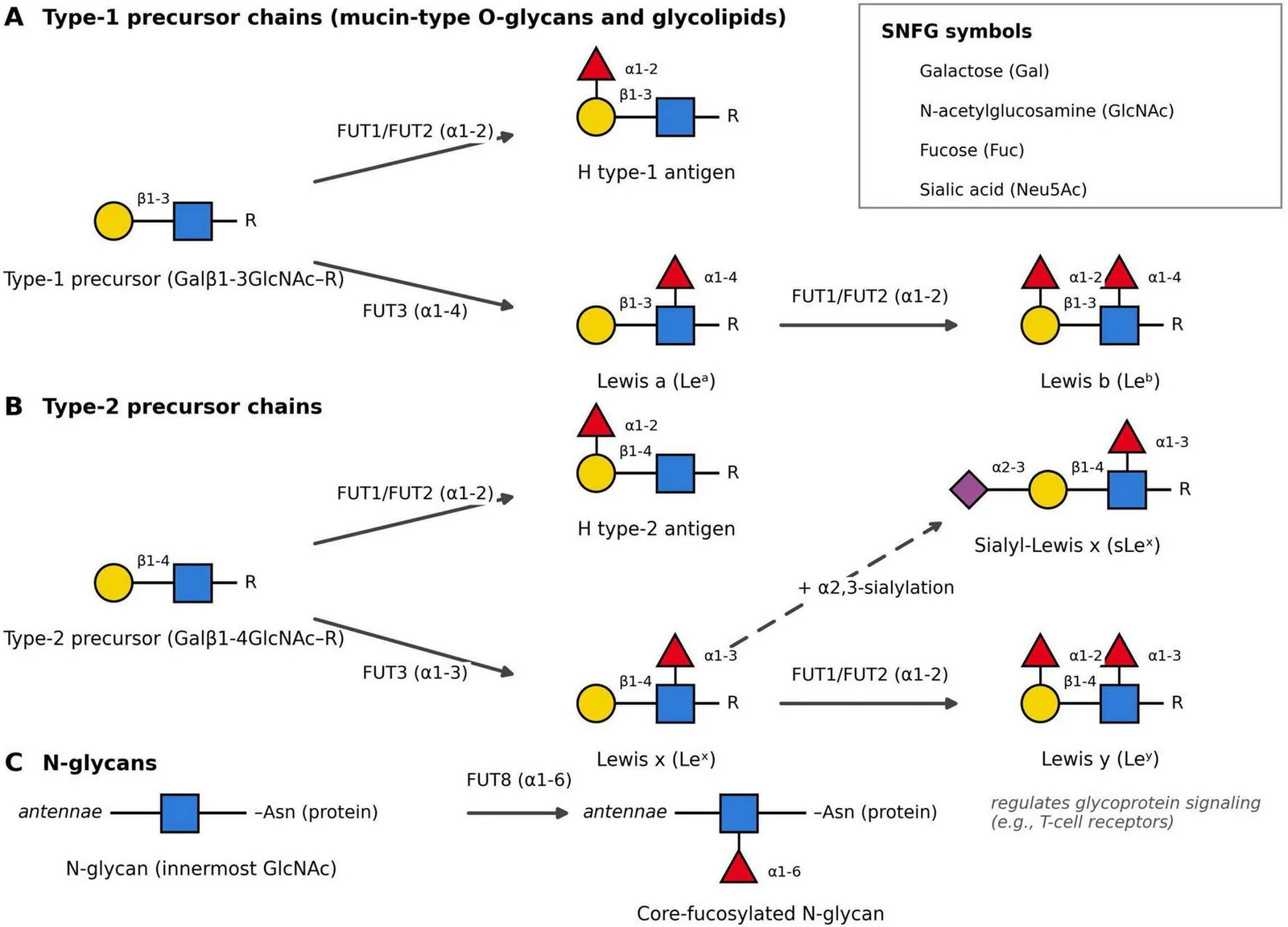
Biosynthesis of major fucosylated glycan structures in the intestinal epithelium. **(A)** On type-1 precursor chains (Galβ1-3GlcNAc; found on mucin-type O-glycans and glycolipids), FUT1 and FUT2 add α1,2-linked fucose to the terminal galactose to form the H type-1 antigen, whereas FUT3 adds α1,4-linked fucose to GlcNAc to form Lewis a (Le^a^); subsequent α1,2-fucosylation of Le^a^ yields Lewis b (Le^b^). **(B)** On type-2 precursor chains (Galβ1-4GlcNAc), FUT1/FUT2 generate the H type-2 antigen, and FUT3 adds α1,3-linked fucose to form Lewis x (Le^x^); further α1,2-fucosylation yields Lewis y (Le^y^), and Le^x^ can also be sialylated to sialyl-Lewis x (sLe^x^). **(C)** On N-glycans, FUT8 adds α1,6-linked fucose to the innermost GlcNAc residue (core fucosylation), thereby regulating glycoprotein signaling, such as T-cell receptor signaling. Glycan symbols follow the Symbol Nomenclature for Glycans (SNFG): yellow circle, galactose (Gal); blue square, N-acetylglucosamine (GlcNAc); red triangle, fucose (Fuc); purple diamond, sialic acid (Neu5Ac). Image created using MedPeer.

Within the intestinal epithelium, fucosylation decorates several major classes of glycan structures (Figure 1). Terminal α1,2-fucosylation, catalyzed by FUT1 and FUT2, modifies galactose residues on mucin-type O-glycans and glycolipids to generate H antigens (H type-1 and H type-2 structures), which also serve as precursors for Lewis-type fucosylated determinants (Terahara et al., 2011; Hu et al., 2022; Kamioka et al., 2022). FUT3 (the Lewis enzyme) adds α1,3- or α1,4-linked fucose to type-1 and type-2 chains, generating Lewis antigens (Le^a^, Le^b^, Le^x^, and Le^y^) that further diversify the intestinal glycan repertoire (Orczyk-Pawiłowicz and Lis-Kuberka, 2020); presented on the apical surface or secreted into the mucus layer, these terminal structures directly interface with the luminal microbiota. By contrast, core α1,6-fucosylation, catalyzed by FUT8, modifies the innermost GlcNAc residue of N-glycans and primarily regulates the signaling functions of the modified glycoproteins, such as T-cell receptors (Fujii et al., 2016). The following sections detail these enzymes, with emphasis on FUT2 as the principal microbiota-responsive fucosyltransferase.

FUT2 is a key intestinal enzyme that α1,2-fucosylates both type-1 and type-2 precursor chains, generating H type-1 and H type-2 structures depending on substrate availability; the emphasis on H type-1 herein reflects its particular relevance to secretor status in the intestinal mucosa—homozygous loss-of-function variants result in the non-secretor phenotype, whereas carriage of at least one functional allele defines the secretor phenotype (Goto et al., 2014; Lei et al., 2022). Most functional studies rely on global *Fut2-*knockout mice, which cannot distinguish cell-type-specific effects (e.g., Paneth cell-derived vs. goblet cell-derived fucosylation) or parse epithelial fucosylation contributions from stromal/immune cell glycosylation. *Fut2* expression is dynamically induced by microbial signals, especially in the ileum (Goto et al., 2014). However, despite high *Fut2* expression in the small intestine, fucosylated glycans are most abundant on mouse Muc2 mucin in the colon (Holmén Larsson et al., 2013). The gut microbiota selectively induces the expression of *Fut2*, while leaving the expression of other FUTs—including *Fut1* and *Fut8*—unaffected (Tsang et al., 2022). Although basal fucosylation on Paneth cells occurs in a microbiota-independent manner (Goto et al., 2014), a functionally distinct FUT2^+^ Paneth cell subpopulation is enriched in the ileum and exhibits elevated production of antimicrobial peptides (Kamioka et al., 2022). Glycocalyx assembly integrates these enzymatic activities: FUT2-mediated α1,2-fucosylation predominantly modifies terminal Galβ1,3GlcNAc (type 1) chains on O-glycans of mucins (e.g., MUC2) and glycolipids. These fucosylated structures are then secreted or presented on the apical surface of intestinal epithelial cells, forming the dense, dynamic glycan layer of the glycocalyx that interfaces with the luminal microbiota. This contrasts mechanistically with extra-intestinal glycocalyx structures, such as the vascular endothelial glycocalyx, which relies predominantly on heparan sulfate proteoglycans (syndecans and glypicans) rather than mucin-type O-glycans. Consequently, the fucosylation patterns discussed herein are specific to the dynamic, secretory environment of the gut epithelium.

FUT1 and FUT2 exhibit a spatially and functionally segregated division of labor: FUT1 catalyzes α1,2-fucosylation on Peyer’s patch M cells and maintains basal fucosylation in Paneth cells, whereas FUT2 mediates microbiota-inducible fucosylation on conventional intestinal epithelial cells (IECs) and on the FUT2^+^ Paneth cell subpopulation (Terahara et al., 2011; Kamioka et al., 2022).

Core fucosylation, the α1,6-linking of fucose to N-glycans by FUT8, is also critical. It is required for T-cell receptor signaling, and *FUT8* expression is increased in IBD. *Fut8*-deficient mice exhibit significant resistance to T cell–mediated experimental colitis (Fujii et al., 2016).

Enzymatic activity that regulates glycan modification is spatially organized along the gastrointestinal axis. Colonic mucus comprises two functionally distinct layers: a thick, fucosylated inner layer (designated b1), primarily secreted by goblet cells in the proximal colon, which physically encapsulates the luminal microbiota; and a thinner, more sulfated outer layer (designated b2), added predominantly by goblet cells in the distal colon (Bergstrom et al., 2020). Both the proximal and distal colon exhibit robust fucosylation of mucins—markedly exceeding levels observed in the small intestine—whereas proximal colonic mucins display higher sialylation, and distal colonic mucins show greater sulfation (Holmén Larsson et al., 2013). The spatial organization of fucosylation—from GDP-fucose synthesis in the cytoplasm to terminal glycan modification in the Golgi and final presentation at the apical membrane—underpins the regional specialization of the intestinal glycocalyx.

### 2.2 Regulation by the immune system

The innate immune system exerts precise, context-dependent regulation of mucosal fucosylation. Type 3 innate lymphoid cells (ILC3s) serve as essential non-hematopoietic regulators that drive epithelial *Fut2* expression in response to microbial and cytokine cues (Goto et al., 2014). This regulation proceeds through two mechanistically distinct pathways: a microbiota-dependent axis driven by IL-22, and a microbiota-independent axis mediated by lymphotoxin (LT) signaling (Goto et al., 2014). Commensal bacteria stimulate ILC3s to secrete IL-22, which in turn drives epithelial *Fut2* expression and is indispensable for the differentiation and functional maturation of the FUT2^+^ Paneth cell subpopulation (Goto et al., 2014; Kamioka et al., 2022).

**FIGURE 2:**
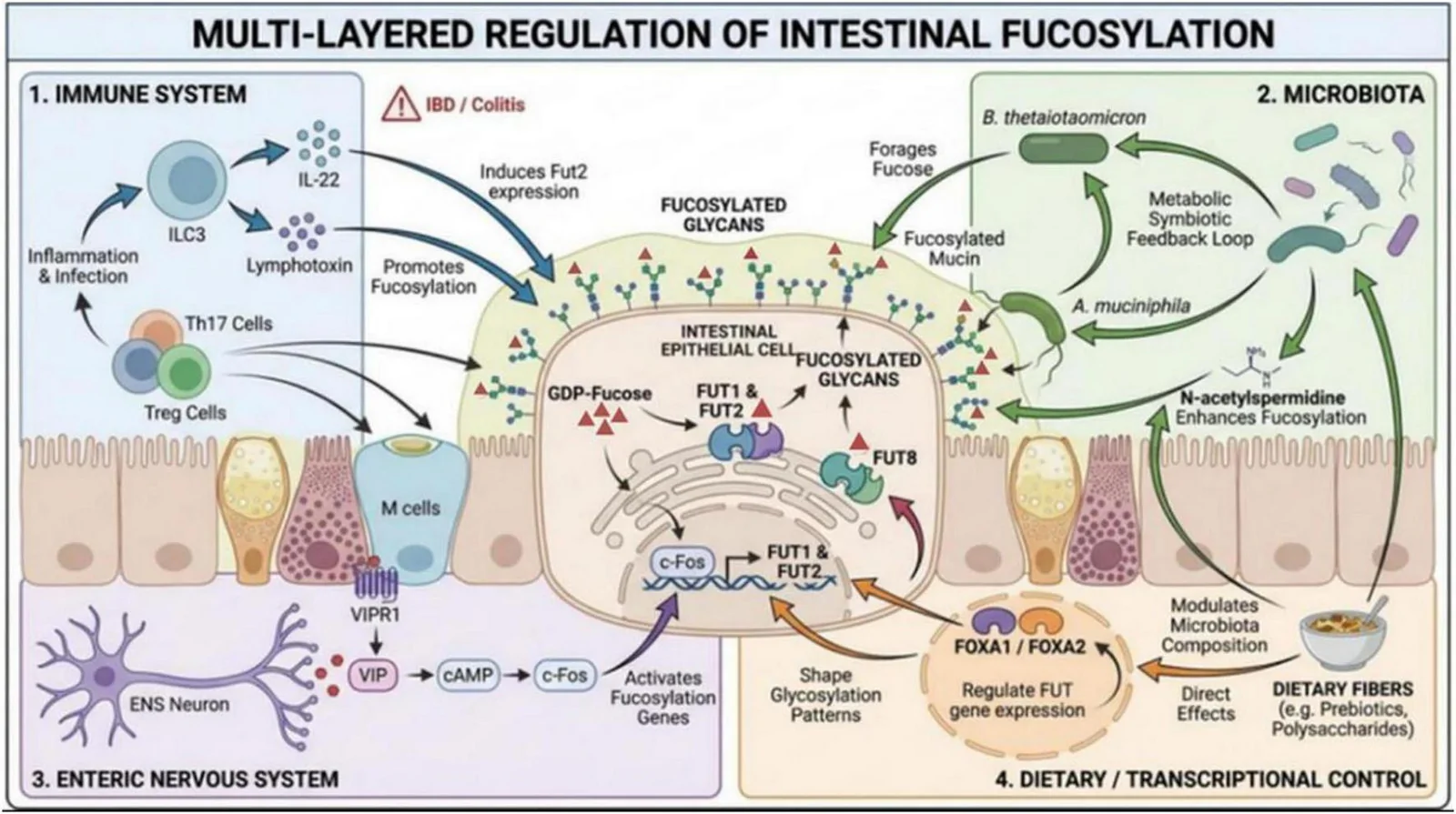
Multifactorial regulation of intestinal fucosylation. Schematic representation of the integrated regulatory network that controls epithelial fucosylation. (1) Immune modulation: Cytokines derived from group 3 innate lymphoid cells (ILC3s; e.g., IL-22) and T helper 17 (Th17) cells (e.g., lymphotoxin) transcriptionally upregulate *Fut2* and other fucosyltransferase enzymes. (2) Microbial crosstalk: Commensal bacteria metabolize fucosylated mucin; subsequently, microbial-derived metabolites (e.g., N-acetylpermidine) provide positive feedback to augment host glycosylation. (3) Neuromodulation: Enteric neurotransmitters, such as VIP, engage the VIPR1–cAMP–c-Fos signaling axis to drive the expression of fucosylation-related genes. (4) Nutritional and transcriptional control: Dietary fibers and prebiotics shape the microbial ecology and modulate the activity of transcription factors (e.g., FOXA1/FOXA2), thereby fine-tuning FUT gene transcription. Collectively, these pathways underscore the intricate interplay among immunological, microbiological, neurological, and nutritional signals in sculpting the intestinal glycome.

This pathway is rapidly engaged during systemic inflammatory stress. Exposure to Toll-like receptor (TLR) ligands initiates a cytokine cascade in which dendritic cells secrete interleukin-23 (IL-23), thereby activating ILC3s to produce IL-22, which in turn induces rapid, transient upregulation of epithelial fucosylation (Pickard et al., 2014). This IL-22–dependent induction of epithelial *Fut2*, mediated through the IL-22 receptor subunit IL-22RA1, enhances colonization resistance against enteric pathogens such as *Salmonella Typhimurium* (Goto et al., 2014; Pham et al., 2014; Figure 2).

### 2.3 Regulation by the microbiota

Intestinal fucosylation is dynamically regulated by a symbiotic feedback loop between the host and the gut microbiota, which drives the transcriptional upregulation of α1,2-fucosyltransferase (*Fut2*) and subsequent biosynthesis of fucosylated glycoconjugates in IECs (Bry et al., 1996). This *Fut2* induction occurs in specific epithelial cell types, including goblet cells and mature enterocytes (Tsang et al., 2022). Commensal bacteria—including *Bacteroides thetaiotaomicron* and segmented filamentous bacteria (SFB)—function as potent, physiologically relevant inducers of host intestinal fucosylation (Bry et al., 1996; Goto et al., 2014). The capacity of *B. thetaiotaomicron* to induce intestinal epithelial fucosylation is mechanistically coupled to its fucose-metabolizing activity (Bry et al., 1996). This symbiotic regulatory loop is nutritionally modulated: low-fiber, purified diets diminish SFB colonization and concurrently attenuate intestinal epithelial fucosylation (Shiratori et al., 2024).

Whether the human *FUT2* genotype (secretor status) shapes the overall fecal microbiota composition remains debated. Large-scale studies found no significant association between secretor status and fecal microbial diversity or composition, contrasting with earlier, smaller reports. This discrepancy likely arises from methodological heterogeneity—including differences in statistical power, uncontrolled confounding variables (e.g., diet, medication use, and geographic origin), and biological sampling source (mucosa-associated versus luminal/fecal microbiota) (Turpin et al., 2018).

Specific commensal bacteria—notably *Akkermansia muciniphila* (*A. muciniphila*)—serve as key modulators of host intestinal fucosylation and mucosal homeostasis. *A. muciniphila* abundance is significantly diminished in *Fut2*-deficient mice, and exogenous administration of this bacterium ameliorates experimental colitis—partly through restoration of α1,2-fucosylated glycoconjugate biosynthesis in the colonic epithelium (Yao et al., 2025). This protective effect is mediated by the bacterial metabolite N-acetylspermidine, which engages host cellular signaling pathways to enhance intestinal epithelial barrier integrity—specifically by promoting membrane localization of fucosylated tight junction proteins (ZO-1 and ZO-2) and concurrently suppressing expression of the pro-inflammatory complement component C3 (Yao et al., 2025). Notably, even heat-inactivated (pasteurized) *A. muciniphila* retains the capacity to modulate intestinal mucin glycosylation *in vitro*—specifically increasing the abundance of sialylated O-linked glycans and high-mannose N-linked glycans (de Ram et al., 2024).

In contrast, enteric pathogens such as *Helicobacter pylori* enzymatically glycosylate their surface adhesins—including BabA and BabB—to augment the structural stability and host epithelial binding affinity of these virulence determinants. The bacterial glycosylation machinery thus constitutes a promising, pathogen-selective therapeutic target for modulating host–microbe interactions (Teng et al., 2022).

Commensal bacteria actively orchestrate their encapsulation within the fucosylated mucus barrier by stimulating Muc2 biosynthesis and secretion from goblet cells in the proximal colon—a regulatory process that is entirely absent in germ-free mice (Bergstrom et al., 2020). This host-derived fucose serves as a critical nutrient source that sustains commensal bacterial populations during periods of dietary nutrient limitation (Pickard et al., 2014). The gut microbiota is indispensable for the postnatal development and α1,2-fucosylation of the functionally specialized FUT2^+^ Paneth cell subtype (Kamioka et al., 2022; Tsang et al., 2022).

### 2.4 Regulation by the enteric nervous system

The ENS exerts direct, neuroanatomically defined control over intestinal α1,2-fucosylation (Ibiza et al., 2016). Consistent with this, subdiaphragmatic vagotomy significantly attenuates *Fut2* expression and α1,2-fucosylated glycoconjugate levels in the ileum (Lei et al., 2022). Enteric neurons that synthesize VIP serve as the primary neural regulators of intestinal α1,2-fucosylation (Talbot et al., 2020). VIP engages its cognate receptor VIPR1 on IECs, thereby activating the ERK1/2–c-Fos signaling axis and driving transcriptional upregulation of the *Fut2* gene (Lei et al., 2022).

The causal role of this VIP-VIPR1 axis is confirmed by *in vivo* administration of VIP agonists/antagonists, chemogenetic manipulation of VIP neurons, and *in vitro* co-culture experiments (Lei et al., 2022). Disruption of the VIP–VIPR1 signaling axis—achieved experimentally via subdiaphragmatic vagotomy—results in diminished intestinal α1,2-fucosylation, leading to dysbiosis characterized by selective depletion of beneficial *Bifidobacterium* species and expansion of opportunistic *Enterococcus faecalis*, thereby exacerbating susceptibility to alcohol-associated liver disease (ALD) (Lei et al., 2022).

### 2.5 Transcriptional and dietary control

Fucosylation is regulated by distinct transcriptional programs. The transcription factors FOXA1 and FOXA2 serve as master regulators of colonic glycosylation; their conditional deletion in the intestinal epithelium disrupts the expression of glycosylation enzymes, induces profound gut microbial dysbiosis, and predisposes mice to spontaneous IBD (Swisa et al., 2024). These programs create regional specialization of mucus properties (Holmén Larsson et al., 2013; Bergstrom et al., 2020). Specific transcription factors, including c-Fos, are induced downstream of ENS signaling and directly bind the *Fut2* promoter (Lei et al., 2022).

Epigenetic regulation also contributes to this process: the *A. muciniphila*–derived metabolite N-acetylspermidine triggers a signaling cascade—involving PIM1 inhibition and HDAC2 upregulation—that enhances expression of the O-glycan chaperone *C1GALT1C1*, thereby promoting fucosylation (Yao et al., 2025).

Dietary factors also serve as potent regulators of mucosal fucosylation. Specifically, low-fiber purified diets suppress fucosylation by diminishing the abundance of fucosylation-inducing bacteria—such as SFB (Shiratori et al., 2024) —whereas dietary supplementation with fucose rescues fucosylation-deficient mice from experimental colitis (Wang et al., 2017). The chemical nature of dietary fiber constitutes a key determinant of mucosal glycosylation patterns: replacing cellulose with wheat dextrin selectively enhances fucosylated O-glycan abundance while concomitantly reducing sialylated O-glycan levels (Gamage et al., 2020). Supplementation with 2’-FL modulates the expression of key glycosyltransferases involved in fucosylation (Paone et al., 2024).

The structural characteristics of dietary polysaccharides precisely govern the compositional profile of mucin O-glycans. Dietary fibers—including pectin and inulin—typically enhance the abundance of fucosylated glycans while concurrently diminishing acidic glycan species, specifically those bearing sialyl or sulfate modifications (Zhao et al., 2023). This modulation is structurally specific: branched fibers (arabinogalactan) promote mono-fucosylated structures, while linear fibers (pectin, inulin) favor di-fucosylated ones, corresponding with increased colonic *Fut2* expression (Zhao et al., 2023).

These diet-induced alterations in mucin O-glycan composition correlate with shifts in specific bacterial taxa: elevated mono-fucosylated glycans are associated with increased abundance of *Parabacteroides distasonis*, whereas di-fucosylated structures correlate with higher colonization levels of *Alistipes* species (Gamage et al., 2020).

## 3 Fucosylated glycans as a niche and nutrient for the gut microbiota

**FIGURE 3:**
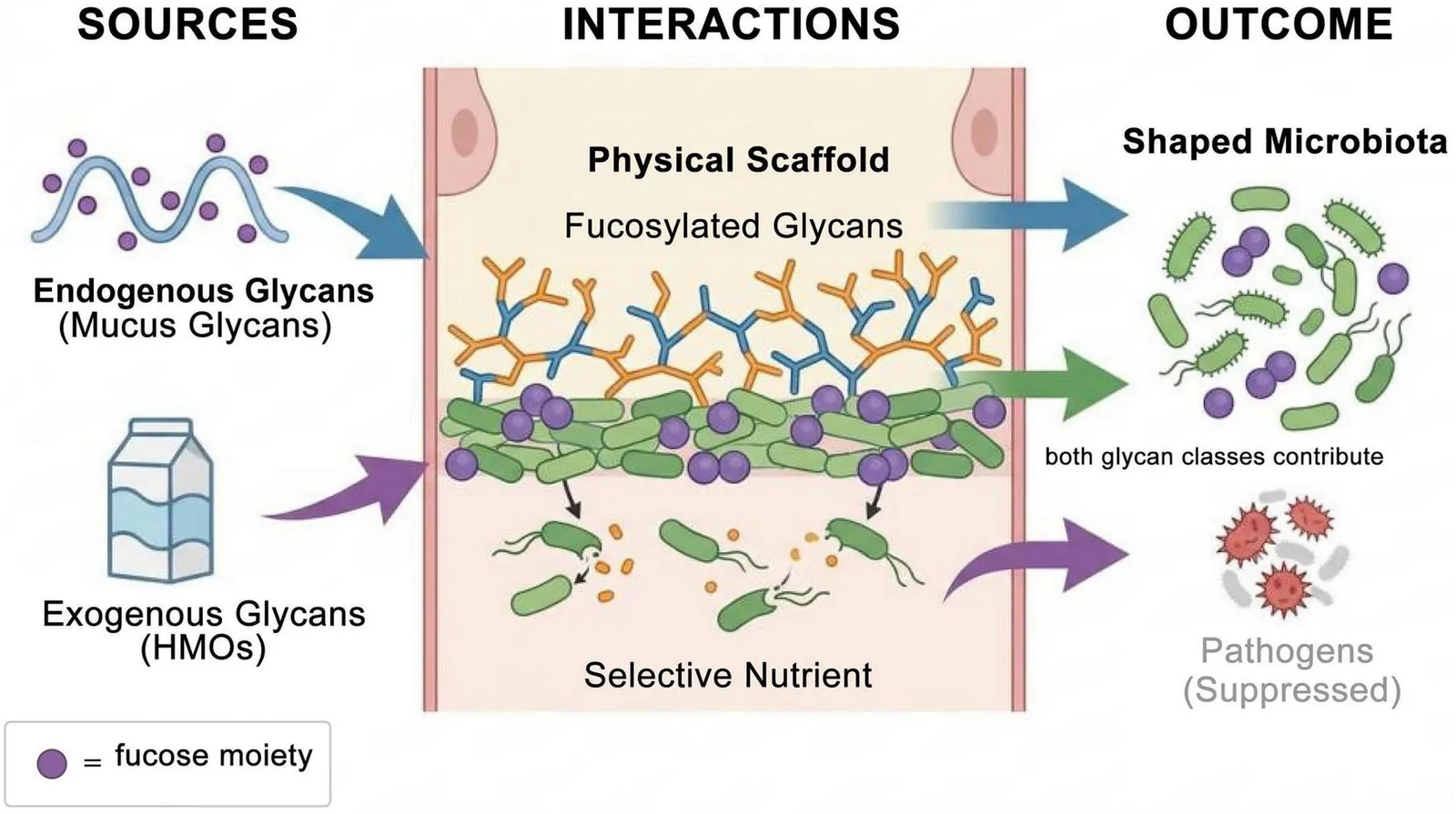
Fucosylated glycans as a niche and nutrient for the gut microbiota. Schematic overview of how endogenous and exogenous fucosylated glycans shape the gut microbiota and suppress pathogens. (Left) Sources: two classes of fucosylated glycans are shown under a unified naming scheme—endogenous glycans (mucus glycans) and exogenous glycans (HMOs). Purple circles denote fucose moieties throughout the diagram. (Middle) Interactions: both glycan classes provide a physical scaffold for microbial attachment and act as selective nutrients that enrich fucose-utilizing commensals. (Right) Outcome: this glycan-mediated interaction network establishes a structured “Shaped Microbiota” and drives the suppression of enteric pathogens. Both endogenous and exogenous fucosylated glycans contribute to pathogen suppression—directly, by acting as soluble decoy receptors that competitively block pathogen adhesion to epithelial glycans (Ruiz-Palacios et al., 2003; Komatsu et al., 2026), and indirectly, by enriching beneficial taxa whose fermentation products (lactate and short-chain fatty acids) and associated antimicrobial activities inhibit pathogen growth (Gibson and Wang, 1994; Yu et al., 2013; Luo et al., 2026).

Fucosylated glycans—carbohydrate structures modified by the deoxyhexose L-fucose—constitute a critical molecular interface mediating host–microbiome interactions (Figure 3). These glycans are delivered to the gut ecosystem via two principal physiological routes: endogenously, as structural constituents of the intestinal mucus layer; and exogenously, as a predominant component of HMOs during infancy. Beyond these two routes, dietary glycoconjugates and fucose-rich polysaccharides (e.g., fucoidans from brown algae and other fucose-containing dietary polysaccharides) provide an additional exogenous source that contributes to the intestinal fucose pool and shapes the gut microbiota, particularly after weaning (Zhao et al., 2023; Lei et al., 2025). This continuous provision of fucose-containing glycans serves a dual physiological role: it provides a structural scaffold that facilitates microbial colonization, and functions as a selective nutrient source that critically shapes the taxonomic composition and functional capacity of the gut microbiota. The capacity to bind and metabolize fucosylated glycans represents a key adaptive trait among numerous gut commensals, thereby driving intricate ecological interactions—including competitive exclusion, metabolic cross-feeding, and host–microbe co-evolution—within the intestinal ecosystem.

### 3.1 Fucosylated mucins as a niche and nutrient source

**FIGURE 4:**
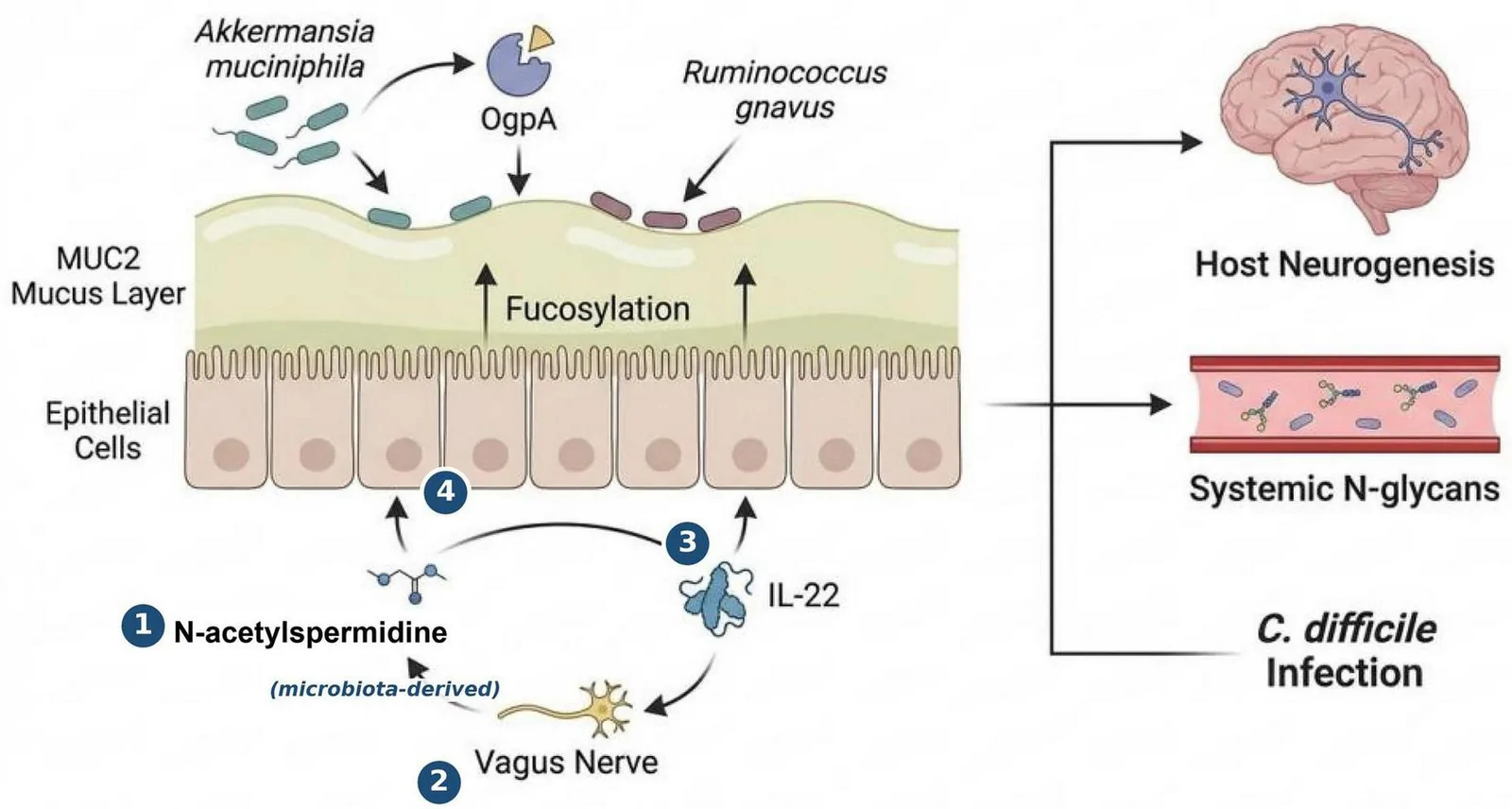
Fucosylated mucins as a niche and nutrient source. Schematic diagram illustrating the interactions among gut microbiota, epithelial cells, and host physiological processes. *A. muciniphila* interacts with the MUC2 mucus layer via OgpA (Trastoy et al., 2020), while *Ruminococcus gnavus* also engages with the mucus layer. These interactions influence epithelial fucosylation. Below the epithelium, a unidirectional signaling axis (numbered circles 1–4) links the microbiota to host fucosylation: (1) microbiota-derived N-acetylspermidine (e.g., from *A. muciniphila*) (Yao et al., 2025) acts on (2) the vagus nerve and enteric neurons (Lei et al., 2022), triggering (3) IL-22 secretion, which in turn induces (4) epithelial fucosylation (Goto et al., 2014). The arrows indicate the direction of signal flow and do not represent a feedback loop. Through this axis, microbial and cellular interactions impact three key host outcomes: host neurogenesis (neural development in the brain) (Coletto et al., 2022), systemic N-glycans (glycan profiles in the circulation) (Monaghan et al., 2019), and susceptibility to *Clostridioides difficile* infection (Monaghan et al., 2019).

The intestinal mucus layer—predominantly composed of the gel-forming mucin MUC2—constitutes a dynamic ecological niche in which glycan structures are both shaped by the resident microbiota and actively shape its composition and functional activity (Cheng et al., 2025). In the murine proximal colon, the resident microbiota stimulates Muc2 synthesis, and the fucosylated O-glycans displayed on this mucin serve dual functional roles—as specific molecular attachment sites for select bacterial taxa and as preferential carbon sources—that collectively modulate gut microbial community structure (Bergstrom et al., 2020) (Figure 4). Critically, commonly used laboratory-grade mucins exhibit markedly low predictive validity for microbial adhesion to native human intestinal mucins, underscoring the imperative to employ biologically relevant, physiologically representative substrates in host–microbe interaction studies (Ringot-Destrez et al., 2018).

Specialist mucin-degrading bacteria—exemplified by *A. muciniphila*—employ dedicated enzymatic machinery, including the O-glycopeptidase OgpA, to cleave host mucin O-glycans and thereby gain access to the underlying protein core. This Zn-dependent metalloprotease recognizes both the polypeptide backbone and proximal O-glycan moieties through a conserved aromatic substrate-binding pocket—formed by residues Y116, F166, W199, and Y236—and cleaves peptide bonds immediately N-terminal to O-glycosylated serine or threonine residues (Trastoy et al., 2020). Similarly, mucin foraging by *Ruminococcus gnavus* exhibits marked strain-level heterogeneity, with distinct strains selectively targeting terminal fucose, sialic acid (N-acetylneuraminic acid), or ABO blood group antigens as carbon and energy sources (Wu et al., 2021; Coletto et al., 2022). In gnotobiotic mice, monocolonization with *Ruminococcus gnavus* leads to a significant depletion of sialylated mucin glycans—including the sialyl-T antigen—thereby remodeling the colonic mucosal glycome (Coletto et al., 2022). Terminal glycans—such as sialic acid (N-acetylneuraminic acid)—serve dual, functionally opposing roles: they act as molecular receptors for enteric pathogens (e.g., *Shigella sonnei*), while simultaneously masking cryptic bacterial attachment sites on the mucin glycoprotein scaffold that are otherwise accessible to commensal strains [e.g., *Escherichia coli (E. coli)* K12], thereby illustrating how structurally defined glycotopes govern microbial colonization dynamics in the gut (Wu et al., 2021). Fucose and sialic acid often occupy adjacent terminal positions on the same mucin O-glycans (e.g., the sialyl-Lewis X epitope carries both modifications). Reduced fucosylation in pathology frequently unmasks underlying sialylated epitopes, shifting microbial binding preference from commensals to pathogens.

Mucosal fucosylation is dynamically regulated through bidirectional host–microbiota crosstalk, wherein microbial metabolites—including *A. muciniphila*-derived N-acetylspermidine—enhance host α1,2-fucosyltransferase activity, thereby establishing a positive feedback loop that reinforces mucosal barrier integrity (Yao et al., 2025). Host factors, including IL-22 signaling (Li and Guo, 2026) and vagus nerve activation (Zhao et al., 2026), also fine-tune this process. Mucin foraging exerts systemic physiological consequences: colonization of mice with *R. gnavus* alters the systemic pool of sialic acid derivatives—including in circulation and the hippocampal region—induces production of gut-derived neuroactive metabolites [e.g., short-chain fatty acids (SCFAs) and tryptophan catabolites], and impairs hippocampal neurogenesis and hippocampus-dependent memory performance (Coletto et al., 2022).

Clinically, dysbiosis of fucose-metabolizing bacterial communities during recurrent *Clostridioides difficile* infection is associated with aberrant systemic N-glycan profiles—including in serum glycoproteins—which revert to homeostatic baselines following fecal microbiota transplantation (Monaghan et al., 2019), and microbiome repair in undernourished children impacts mucin-producing goblet cells; similarly, microbiome-directed therapeutic interventions in undernourished children restore goblet cell density and enhance mucin biosynthesis and secretion (Wang et al., 2025).

### 3.2 Fucosylated HMOs in shaping the infant gut

**FIGURE 5:**
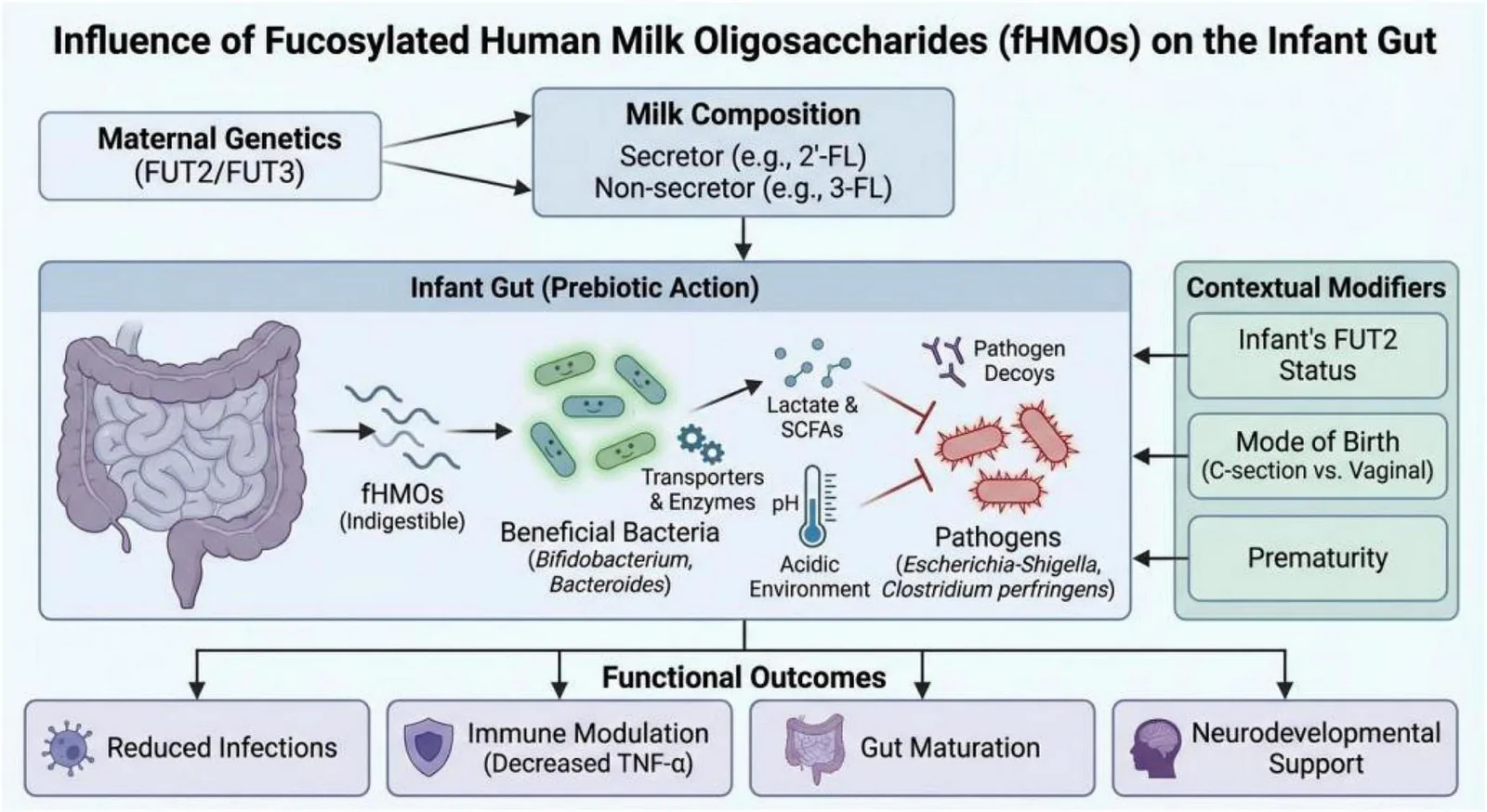
Influence of fHMOs on the infant gut. Schematic overview of how fHMOs shape the infant gut through integrated maternal, microbial, and environmental pathways. Maternal *FUT2/FUT3* genetics dictate milk fHMO profiles—secretor milk is enriched in α1,2-fucosylated HMOs such as 2’-fucosyllactose (2’-FL), which are undetectable in non-secretor milk; 3’-FL is present in both secretor and non-secretor milk, differing in relative abundance. Indigestible fHMOs pass into the infant gut, where they function as prebiotic substrates to promote beneficial bacteria (*Bifidobacterium*, *Bacteroides*), driving production of lactate and SCFAs that lower gut pH, while also acting as “pathogen decoys” by mimicking host glycans to bind and inhibit colonization by pathogens like *Escherichia-Shigella* and *Clostridium perfringens*. These interactions are modulated by contextual factors including the infant’s own *FUT2* status, mode of birth (cesarean vs. vaginal delivery), and prematurity. Collectively, these mechanisms yield key health outcomes: reduced infection risk, immune modulation (e.g., decreased pro-inflammatory TNF-α), enhanced gut maturation, and support for neurodevelopment, illustrating the multi-level role of fHMOs in infant physiology.

**FIGURE 6:**
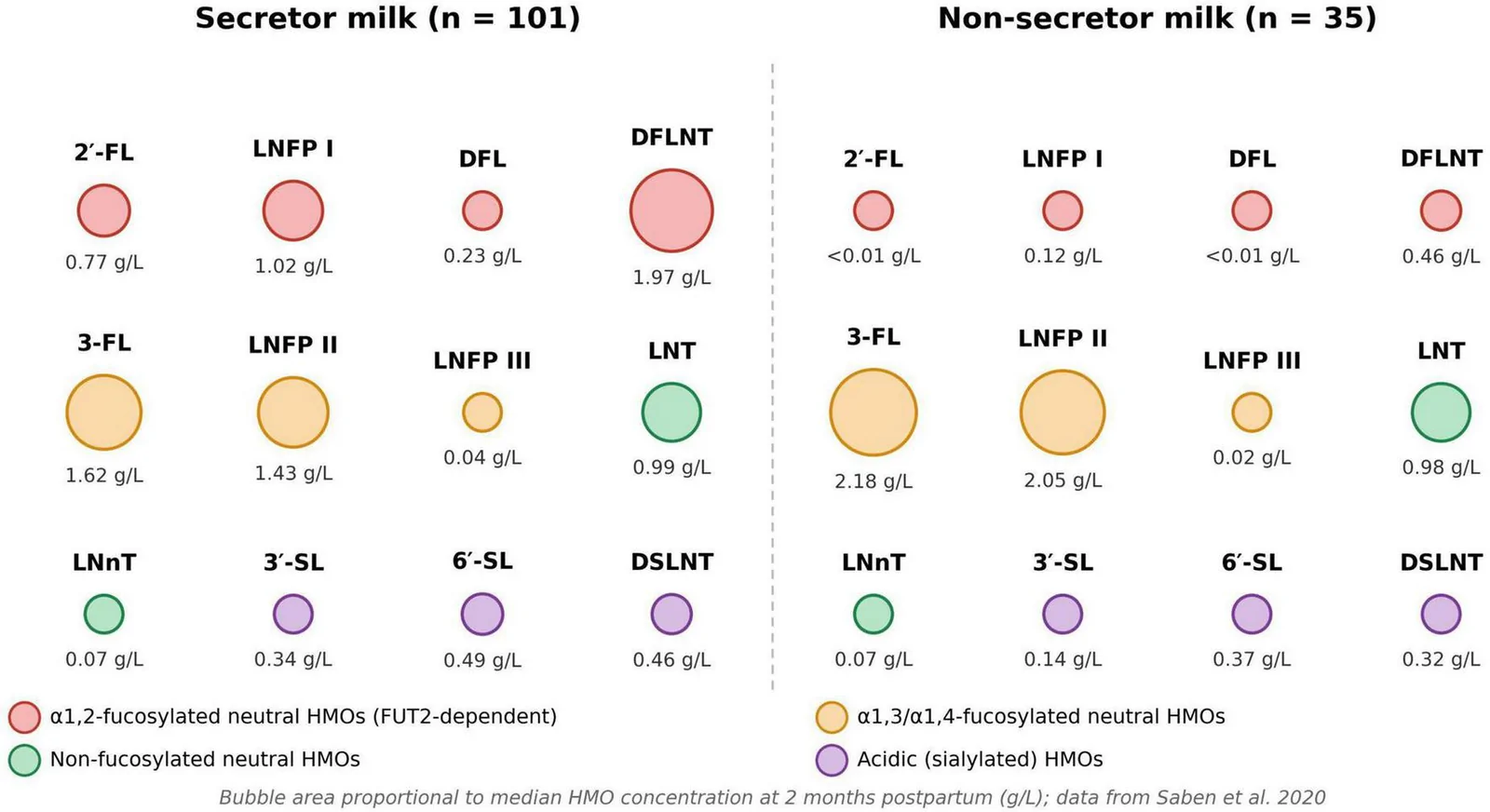
Relative abundance of fHMOs in secretor versus non-secretor milk. Bubble area is proportional to the median concentration of each HMO in milk collected at 2 months postpartum (values in g/L, converted from nmol/mL; data Saben et al. (2020), *n* = 101 secretor and *n* = 35 non-secretor mothers). Milk from secretor mothers (functional *FUT2* allele; left) is characterized by high concentrations of α1,2-fucosylated neutral HMOs (red)—notably (2’-FL, lacto-N-fucopentaose I (LNFP I), difucosyllactose (DFL), and difucosyllacto-N-tetraose (DFLNT)—which are present only at trace levels in non-secretor milk (right). Non-secretor milk instead contains higher concentrations of α1,3/α1,4-fucosylated neutral HMOs (orange), such as 3-fucosyllactose (3-FL) and LNFP II, whereas non-fucosylated neutral HMOs [green; e.g., lacto-N-tetraose (LNT) and lacto-N-neotetraose (LNnT)] and acidic, sialylated HMOs (purple) occur at comparable levels in both groups. Note that 3-FL is present in both secretor and non-secretor milk, differing in relative abundance rather than in presence or absence. Red, α1,2-fucosylated neutral HMOs (FUT2-dependent); orange, α1,3/α1,4-fucosylated neutral HMOs; green, non-fucosylated neutral HMOs; purple, acidic (sialylated) HMOs. Data from Saben et al. (2020); figure concept adapted from Walsh et al. (2020). Image created using MedPeer.

During infancy, fHMOs and fucosylated human milk glycoproteins (fHMGs) are a dominant force in shaping the nascent gut microbiota (Orczyk-Pawiłowicz and Lis-Kuberka, 2020) (Figure 5). These complex carbohydrates, which are largely indigestible by the infant’s own digestive system due to a lack of the required secretory or brush-border glycosidases (Dallas et al., 2012), act as a powerful prebiotic, selectively fueling the growth of beneficial bacteria (Luo et al., 2026) (Table 2). This glycosylation also confers proteolytic resistance to the protein backbone of HMGs, thereby preserving their structural integrity and functional bioactivity until they reach the distal gut (Dallas et al., 2012). This selective fermentation not only enriches beneficial commensal taxa but also actively suppresses the colonization and expansion of pathobionts—including *Escherichia–Shigella* (Luo et al., 2026) and *Clostridium perfringens* (Yu et al., 2013)—through competitive exclusion and modulation of luminal pH and redox potential. This inhibition is largely indirect; fHMOs are fermented by bifidobacteria into lactate and SCFAs, and the resulting acidic environment contributes substantially to the suppression of these pathogens (Yu et al., 2013). Acidification is unlikely to be the sole mechanism, however: co-culture experiments have shown that *Bifidobacterium infantis* inhibits *E. coli* and *Clostridium perfringens* through effects not fully explained by acid production, and several bifidobacterial species secrete antimicrobial compounds with broad-spectrum activity against enteric pathogens (Gibson and Wang, 1994). In these mixed microbial communities, major fHMOs are rapidly and extensively metabolized, with > 90% of 2’-FL and lactodifucotetraose (LDFT) being consumed within the experimental timeframe (Yu et al., 2013). This suppressive effect also extends to structurally simpler fHMO-derived building blocks: *in vitro* fermentation experiments demonstrate that fucosyl-α1,6-N-acetylglucosamine (6’-FN), a disaccharide naturally present in host N-glycans, significantly reduces the relative abundance of members of the Enterobacteriaceae family (Rubio-Del-Campo et al., 2020). This protective effect is corroborated by clinical evidence demonstrating that infant formula supplemented with 2’-FL is associated with a reduced incidence of gastrointestinal infections and a lower requirement for antibiotic treatment (Komatsu et al., 2026). This is further corroborated by findings from a large prospective cohort study of exclusively breastfed infants in Indonesia, which revealed that infants born to secretor-positive mothers exhibited a significantly lower prevalence of cough compared with those born to secretor-negative mothers (Wang et al., 2023). This protective association with reduced risk of respiratory illness was prospectively validated in a cohort of 240 exclusively breastfed infants in Bangladesh, wherein maternal secretor-positive status was associated with a 34% lower incidence rate of ARIs during the first 6 months of life (Binia et al., 2021). The composition of HMOs and HMGs is predominantly governed by maternal genetic factors, with the *FUT2* gene representing a key determinant. This genetic variation functions within a highly coordinated biosynthetic network; computational modeling indicates that the combinatorial activity of a core set of only 11 glycosyltransferases accounts for over 90% of the more than 200 structurally distinct HMO isomers identified to date (McDonald et al., 2022). Mothers homozygous or heterozygous for a functional *FUT2* allele (“secretors”) produce human milk enriched in α1,2-fucosylated HMOs, including 2’-FL; in contrast, mothers homozygous for loss-of-function *FUT2* alleles (“non-secretors”) lack detectable α1,2-fucosylated HMOs and instead secrete milk with relatively higher concentrations of alternative fucosylated isomers, such as 3-fucosyllactose (3’-FL) (Figure 6). Maternal genotype at the Lewis locus (*FUT3*) further modulates HMO composition, yielding at least four distinct HMO phenotypic profiles defined by the combined secretor (*FUT2*) and Lewis (*FUT3*) status: secretor-positive/Lewis-positive (Se^+^/Le^+^, ∼70% of the population), secretor-positive/Lewis-negative (Se^+^/Le^–^, ∼9%), secretor-negative/Lewis-positive (Se^–^/Le^+^, ∼20%), and secretor-negative/Lewis-negative (Se^–^/Le^–^, ∼1%) (Orczyk-Pawiłowicz and Lis-Kuberka, 2020). This genetic polymorphism establishes functionally distinct nutritional milieus for the infant; for example, total HMO concentration in colostrum is approximately two-fold higher in secretor-positive mothers compared with secretor-negative mothers (9.7 g/L versus 5.2 g/L) (Orczyk-Pawiłowicz and Lis-Kuberka, 2020). This nutritional milieu is further modulated by lactation stage: quantitative analyses reveal that the extent of α1,2-fucosylation and α1,6-fucosylation in HMGs declines to approximately 42 and 49% of their respective concentrations in early colostrum by the mature milk stage (Orczyk-Pawiłowicz and Lis-Kuberka, 2020). Although human milk contains endogenous glycosidases—including α-L-fucosidase—their enzymatic activity is negligible under physiological conditions and insufficient to meaningfully modify glycan structures prior to microbial processing in the infant gut (Dallas et al., 2012). Differential intestinal absorption of specific HMOs further modulates their systemic bioavailability; pronounced differences have been documented even among structural isomers—exemplified by the contrasting pharmacokinetic fates of 3’-sialyllactose (3’-SL) and its isomer 6’-sialyllactose (6’-SL): 3’-SL is efficiently absorbed and excreted unchanged in urine, whereas 6’-SL remains largely unabsorbed in the gastrointestinal tract, indicating that glycosidic linkage specificity governs systemic exposure (Underwood et al., 2015). This nutritional milieu is further shaped by the infant’s *FUT2* genotype, which determines the fucosylation status of the intestinal mucosa and thereby provides an endogenous source of fucose for microbial metabolism (Thorman et al., 2023). However, the host’s nutritional status can also profoundly alter the intestinal glycan landscape. For example, a murine model of postnatal growth restriction induced by maternal undernutrition demonstrated that colonic mucosal fucosylation was preserved, whereas sialylation—particularly on O-glycans—was significantly attenuated (Ley et al., 2019). This specific perturbation of the intestinal glycome was associated with increased intestinal permeability, a pro-inflammatory mucosal milieu, and microbial dysbiosis—characterized by elevated relative abundance of *Escherichia–Shigella*—culminating in heightened susceptibility to chronic colitis in adulthood (Ley et al., 2019).

**TABLE 2:** Major biological functions and clinical effects of fucosylated HMOs (fHMOs).

Function/effect	Key findings and mechanisms	Citation (PMID)
Prebiotic enrichment of beneficial bacteria	Largely indigestible fHMOs/ human milk glycoproteins (HMGs) act as a dominant prebiotic force, selectively fueling beneficial bacteria (e.g., *Bifidobacterium*); the major fHMOs 2’-FL and LDFT are avidly consumed ( > 90% utilization) by the gut microbiota.	(Yu et al., 2013; Orczyk-Pawiłowicz and Lis-Kuberka, 2020; Luo et al., 2026)
Suppression of pathogens	Microbial fermentation of fHMOs produces lactate and SCFAs, lowering gut pH and indirectly suppressing *Escherichia-Shigella* and *Clostridium perfringens*; the disaccharide 6FN significantly reduces the abundance of Enterobacteriaceae *in vitro*.	(Yu et al., 2013; Rubio-Del-Campo et al., 2020; Luo et al., 2026)
Protection of milk glycoproteins	Glycosylation of HMGs protects the protein backbone from premature proteolysis, preserving bioactivity until it reaches the lower gut.	(Dallas et al., 2012)
Genetic determinants of milk fucosylation	*FUT2* determines maternal “secretor” status, yielding milk rich in α1,2-fucosylated HMOs such as 2’-FL; *FUT3* (Lewis gene) combines with *FUT2* to define at least four HMO composition groups; secretor mothers have nearly double the colostrum HMO concentration (9.7 g/L vs. 5.2 g/L).	(Orczyk-Pawiłowicz and Lis-Kuberka, 2020; McDonald et al., 2022)
Lactation-stage dynamics	The fucosylation of HMGs in mature milk drops to approximately half the level seen in early colostrum.	(Orczyk-Pawiłowicz and Lis-Kuberka, 2020)
Clinical effects in infants	2’-FL supplementation in infant formula reduces the incidence of gastrointestinal infections and the need for antibiotics; infants breastfed by secretor mothers show a significantly lower prevalence of cough and acute respiratory infections (ARIs).	(Binia et al., 2021; Wang et al., 2023; Komatsu et al., 2026)

fHMO, fucosylated human milk oligosaccharide; HMG, human milk glycoprotein; HMO, human milk oligosaccharide; 2’-FL, 2’-fucosyllactose; LDFT, lactodifucotetraose; 6FN, fucosyl-α1,6-N-acetylglucosamine; SCFA, short-chain fatty acid; FUT, fucosyltransferase; ARI, acute respiratory infection; PMID, PubMed identifier.

This selective pressure preferentially enriches specific bacterial taxa, most prominently members of the genus *Bifidobacterium*. Indeed, *in vitro* fermentation of a complex HMO mixture with infant fecal microbiota consistently induces a significant and reproducible expansion of *Bifidobacterium* populations (Yu et al., 2013); moreover, HMOs exhibit exceptional prebiotic efficacy—demonstrating superior capacity to lower culture pH relative to an equimolar dose of fructooligosaccharide (FOS), a well-characterized reference prebiotic (Yu et al., 2013). Fermentation of individual fucosylated fHMOs, such as 2’-FL, also supports the proliferation of additional beneficial bacterial genera—including *Lactobacillus* and *Enterococcus* (Luo et al., 2026). However, this prebiotic effect is not consistently observed *in vivo*—particularly in vulnerable populations such as preterm infants—whose gut microbiota frequently exhibits a pronounced depletion of canonical HMO-utilizing taxa, including *Bifidobacterium* and *Bacteroides* species (Underwood et al., 2015). This physiological challenge was explicitly demonstrated in a clinical study of preterm infants (median gestational age: 28 weeks) administered *Bifidobacterium breve* as a probiotic intervention. The cohort exhibited heterogeneous gut colonization patterns, wherein successful microbial engraftment—termed “responders”—was robustly associated with the HMO profile of the ingested milk; specifically, milk consumed by responders contained a significantly higher relative abundance of fucosylated HMOs and a correspondingly lower abundance of non-fucosylated (i.e., undecorated) HMOs (Underwood et al., 2017). Conversely, infants classified as “non-responders”—defined by the absence of sustained *Bifidobacterium breve* engraftment—exhibited significantly elevated fecal abundances of Enterobacteriaceae and *Clostridiaceae* (Underwood et al., 2017). In this high-risk cohort of preterm infants, maternal secretor status did not predict probiotic response—highlighting that the total fucosylated HMO content, rather than the mere presence of α1,2-fucosylated structures, serves as a critical determinant of *Bifidobacterium* engraftment (Underwood et al., 2017). For example, a large-scale cohort study of Indonesian infants—with exclusive breastfeeding rigorously confirmed by maternal recall supplemented with biomarker validation (e.g., urinary LNT)—found no statistically significant difference in the genus-level relative abundance of *Bifidobacterium* between infants born to secretor versus non-secretor mothers (Wang et al., 2023). In this high-risk cohort of preterm infants, maternal secretor status was associated with a statistically discernible trend toward reduced relative abundance of the phylum Proteobacteria—including pathogenic members of the order *Enterobacterales*—and increased relative abundance of the phylum Firmicutes (Underwood et al., 2015). This observation is echoed by findings from a large European cohort of over 200 breastfed infants, which—despite confirming that fHMO profiles are strongly determined by maternal secretor and Lewis status—found that the strongest associations with infant *Bifidobacterium* abundance involved two non-fucosylated HMOs: 6’-sialyllactose (6’-SL), associated with lower relative abundance, and lacto-N-hexaose (LNH), a common branched-core HMO structure, associated with higher relative abundance (McDonald et al., 2022; Barnett et al., 2023). Collectively, these large-scale cohort studies indicate that the influence of maternal secretor status on infant gut microbiota composition is modulated by multiple contextual factors—including geographic region, host genetic and demographic background, infant gestational age and postnatal developmental stage, and the presence of other structurally distinct, biologically active HMOs. For instance, pronounced geographic variation in HMO composition has been documented: the concentration of 3’-FL in breast milk from Swedish mothers was more than fourfold higher than that observed in mothers from rural Gambia (Orczyk-Pawiłowicz and Lis-Kuberka, 2020). Furthermore, 2’-FL exhibits evidence of cross-regulatory interactions with other HMOs, demonstrating positive associations with LNnT and inverse associations with LNT—a pattern that may underlie certain complex *in vivo* associations observed across cohort studies (Orczyk-Pawiłowicz and Lis-Kuberka, 2020). A key interacting factor is the mode of birth. This context-dependency is underscored by a study demonstrating that, among infants delivered vaginally—whose gut microbiota exhibit minimal perinatal perturbation—maternal secretor status exerted no statistically significant effect on overall microbial community structure (Korpela et al., 2018). In contrast, the presence of α1,2-fucosylated HMOs in breast milk from secretor mothers is associated with attenuation of cesarean delivery–associated microbial dysbiosis (Korpela et al., 2018; Tonon et al., 2021). This vulnerability is particularly pronounced in infants born to non-secretor mothers, among whom cesarean delivery–associated microbial dysbiosis is significantly exacerbated (Korpela et al., 2018). This combined effect leads to a profound depletion of bifidobacteria, particularly *Bifidobacterium bifidum* and *Bifidobacterium breve*, and a concurrent bloom of *Enterococcus* species, while also reducing the species-level diversity within the *Bifidobacterium* and *Bacteroides* genera (Korpela et al., 2018). While the milk of secretor mothers helps normalize the overall abundance of the *Bifidobacterium* genus in C-section infants to levels seen in vaginally born infants, this restorative effect is partial (Tonon et al., 2021). For instance, one study showed that cesarean-born infants of secretor mothers still harbored significantly lower levels of *Bacteroides* and a reduced prevalence of *Bifidobacterium longum*, while having higher abundances of *Akkermansia* and the proteobacterium *Kluyvera* (Tonon et al., 2021). This increase in *Akkermansia* has been independently corroborated, lending support to the hypothesis that *A. muciniphila* may preferentially utilize fHMOs in the absence of its dominant bacterial competitors—a condition associated with cesarean delivery (Korpela et al., 2018). Indeed, an exclusive emphasis on maternal genetics may obscure a more influential determinant—the infant’s secretor status. A study of exclusively breastfed infants in a US-based cohort revealed that the infant’s secretor status—not the mother’s—was more robustly associated with gut microbial composition (Thorman et al., 2023). This study further stratified infants according to secretor phenotype into “non-secretor,” “low-secretor,” and “full-secretor” groups—each corresponding to a distinct gut microbial enterotype. Full-secretor infants exhibited higher gut alpha diversity, and the enterotypes were distinguished by the differential dominance of specific *Bifidobacterium* species: non-secretor infants were enriched in *Bifidobacterium breve*, whereas low-secretor infants were enriched in *Bifidobacterium longum* (Thorman et al., 2023). This species-level selectivity is further supported by *in vitro* studies using fHMO structural analogs, which demonstrate that fucosyl-α1,3-GlcNAc (3FN)—a fucosylated disaccharide present in non-secretor human milk and structurally related to 3-FL—significantly enhances the growth of *B. breve* while concurrently inhibiting that of *Bifidobacterium bifidum* (Rubio-Del-Campo et al., 2020). This suggests that the infant’s endogenous mucosal fucosylation—not the maternal milk fHMO profile alone—drives species-level selectivity within the *Bifidobacterium* genus, potentially accounting for the absence of a consistent genus-level effect in larger cohorts. However, the clinical implications of the infant’s genotype are complex: in the Bangladeshi cohort, maternal secretor status was protective, whereas secretor-positive infants paradoxically exhibited a significantly higher risk of ARIs during early infancy (Binia et al., 2021).

However, an exclusive focus on bifidobacteria may be overly narrow, as other prominent gut commensals—particularly *Bacteroides* species—are now recognized as significant consumers of HMOs (Kijner et al., 2022). Several *Lactobacillus* species are also commonly detected in breastfed infants and encode glycosyl hydrolases (including α-L-fucosidases) and carbohydrate transport systems that enable the utilization of at least a subset of HMOs and their constituent sugars (Rodríguez-Díaz et al., 2012; Zúñiga et al., 2021). This is particularly evident in immature gut ecosystems—such as that of preterm infants—in which Proteobacteria and Firmicutes frequently predominate at the phylum level. In this context, specific HMO groups exhibit strong and opposing correlations with these dominant phyla: for instance, in preterm infants, the relative abundance of fucosylated HMOs in human milk is negatively correlated with Proteobacteria but positively correlated with Firmicutes, whereas non-fucosylated, non-sialylated HMOs display the opposite correlation pattern (Underwood et al., 2015). Similarly, *A. muciniphila*—a mucin-degrading specialist and another key member of the infant gut microbiota—utilizes HMOs by exploiting the structural similarity between mucins and milk glycans, a capability that is highly strain-dependent and varies across its four known phylogroups (Luna et al., 2022). Although all *Akkermansia* strains possess a genomic toolkit for HMO deconstruction, their efficiency varies with respect to fucosylated HMOs: for instance, a strain from the AmIV phylogroup—which harbors a higher copy number of α-fucosidase genes—exhibits significantly greater growth on 2’-FL and 3-FL compared with strains from other phylogroups (Luna et al., 2022). However, the effect may be context-dependent: in a humanized mouse model colonized with infant fecal microbiota, supplementation with the fucosylated disaccharides 3FN and fucosyl-α1,6-GlcNAc resulted in the complete depletion of *Akkermansia*, suggesting that certain fucosylated HMO structures can exert selective negative pressure on this genus (Rubio-Del-Campo et al., 2021). The selective pressures of these simple structures appear to be highly context-dependent, as *in vitro* fermentation of these same disaccharides with infant fecal microbiota revealed a different pattern of modulation: 6FN specifically reduced the abundance of the Enterobacteriaceae family, while 3FN promoted the growth of the *Lactobacillus* genus and suppressed the *Blautia coccoides* group (Rubio-Del-Campo et al., 2020). This broader perspective is robustly supported by metagenomic analyses of fecal samples from breastfed infants, which have revealed a vast and functionally diverse repertoire of α-L-fucosidases—the key enzymes responsible for fucose liberation—distributed across dominant bacterial genera, including *Bacteroides*, *Phocaeicola*, *Ruminococcus*, and *Streptococcus (Moya-Gonzálvez et al., 2022)*. The same humanized mouse study provided *in vivo* support, showing that these simple fucosylated disaccharides significantly increased the abundance of *Ruminococcus* and that 3FN specifically promoted the growth of *Coprococcus* (Rubio-Del-Campo et al., 2021), further highlighting the different outcomes between *in vivo* mouse models and *in vitro* human fecal cultures. Functional characterization of these enzymes reveals a remarkable diversity in stereospecificity and regiospecificity, with distinct α-L-fucosidases required to hydrolyze the α1, 2-, α1, 3-, α1, 4-, and α1,6-glycosidic linkages present in both fHMOs and HMGs (Dallas et al., 2012; Moya-Gonzálvez et al., 2022). This enzymatic diversity highlights the infant gut microbiota’s broad functional capacity to metabolize fucose derived from both dietary and host-derived sources. This functional capacity exhibits strain-level specificity, as evidenced by substantial inter-strain variation in fucose utilization among distinct *Bacteroides* isolates (Kijner et al., 2022). In contrast to the highly specialized HMO-utilization gene clusters characteristic of *Bifidobacterium* species, certain *Bacteroides* strains deploy a functionally distinct metabolic strategy for HMO processing. For instance, transcriptomic profiling of *Bacteroides dorei* cultivated on 2’-FL identified significant upregulation of genes encoding enzymes from 17 distinct glycoside hydrolase (GH) families (Kijner et al., 2022). This transcriptional response is broadly conserved across diverse HMO substrates, characterized by coordinated upregulation of multiple GH families commonly shared among *Bacteroides* strains—a strategy functionally distinct from the highly specialized, substrate-specific gene clusters deployed by *Bifidobacterium* species (Kijner et al., 2022). Furthermore, the capacity to metabolize fHMOs is not universal among bifidobacteria and can be highly strain-dependent even within a single species. For instance, certain strains of *Bifidobacterium longum*—such as ATCC 15697 and JCM 7007- exhibit high metabolic efficiency in utilizing 2’-FL, 3-FL, and LDFT, thereby generating substantial quantities (Yu et al., 2013). However, even these closely related strains exhibit distinct metabolic preferences; for example, one strain exhibits higher efficiency in the utilization of 2’-FL, whereas another demonstrates superior capacity for the biotransformation of 3-FL (Yu et al., 2013). This functional specialization is further substantiated by a comparative analysis of 21 phylogenetically distinct *Bifidobacterium pseudocatenulatum* strains isolated from healthy infants, revealing that only four strains possess the capacity to catabolize both 2’-FL and 3-FL (Sanchez-Gallardo et al., 2024). Certain strains, like *Bifidobacterium longum* subsp. *infantis*, have evolved sophisticated molecular machinery to capitalize on this nutrient source. Detailed molecular investigations have identified two phylogenetically related yet functionally distinct ATP-binding cassette (ABC) transporters in *Bifidobacterium infantis*—designated FL transporter-1 and FL transporter-2—that are specifically adapted for the uptake of fHMOs (Sakanaka et al., 2019). These transporters exhibit overlapping yet functionally divergent substrate specificities: although both mediate efficient uptake of 2’-FL and 3-FL, FL transporter-2 displays an expanded substrate spectrum that additionally encompasses structurally complex fucosylated HMOs, including LDFT. This ability to utilize both 2’-FL and 3-FL is a critical adaptation, allowing *Bifidobacterium infantis* to thrive on milk from both secretor and non-secretor mothers. The pivotal role of these transporters as determinants of ecological fitness was confirmed in competitive colonization assays: *in vitro* growth competitions revealed that wild-type *Bifidobacterium infantis* significantly outcompeted a double-knockout mutant strain- deficient in both FL transporter-1 and FL transporter-2- when cultured on a complex HMO mixture (Sakanaka et al., 2019).

Structural and phylogenetic analyses further support a compelling model of host–microbe coevolution, indicating that FL transporter-2 likely arose from FL transporter-1 through gain-of-function mutations that broadened its ligand-binding specificity to accommodate the most abundant fHMOs (Sakanaka et al., 2019). A parallel evolutionary trajectory of functional expansion is evident across related bifidobacterial species: in *B. pseudocatenulatum*, the acquisition of an additional GH family 29 (GH29) α-fucosidase—integrated within its fHMO utilization gene cluster—confers enhanced metabolic capacity toward structurally complex fHMOs, including lacto-N-fucopentaose II (LNFPII), which are poorly degraded by strains encoding only a GH95 α-fucosidase (Sanchez-Gallardo et al., 2024). Large-scale genomic analysis of the species further underscores that genes for fHMO degradation are variably present, highlighting their role as specialized adaptive traits rather than core metabolic functions (Sanchez-Gallardo et al., 2024). Consequently, the efficient uptake and intracellular processing of fHMOs—mediated by these strain-specific transporters and GHs—constitutes a key molecular determinant of successful colonization and sustained persistence of probiotic bifidobacteria, including *Bifidobacterium infantis* and selected strains of *B. pseudocatenulatum*, in the infant gastrointestinal tract (Sanchez-Gallardo et al., 2024; Wang et al., 2024). This selective utilization was further demonstrated in a cohort of preterm infants administered a *B. breve* probiotic supplement: longitudinal analysis revealed a statistically significant correlation between the temporal dynamics of fecal bifidobacterial abundance and the depletion of specific structurally complex fHMOs, including LDFT and the lacto-N-fucopentaose (LNFP) isomers I, II, and III (Underwood et al., 2017).

The functional consequences of this selective process are clearly reflected in the infant fecal metabolome and extend to systemic physiological effects. For example, infants born to secretor-positive mothers display distinct fecal metabolic profiles—characterized by significantly reduced levels of succinate, glycine, and related amino acid derivatives, alongside elevated concentrations of 1,2-propanediol (1,2-PD), a microbial fermentation product derived from fucose metabolism (Wang et al., 2023). However, fucose fermentation can also serve as a potent driver of SCFA biosynthesis. In a humanized murine model, supplementation with 3’-FL—a fHMO constituent—significantly elevated colonic concentrations of both butyrate and acetate; notably, butyrate was undetectable under baseline conditions and became quantifiable only following 3’-FL administration (Rubio-Del-Campo et al., 2021). This butyrogenic effect is not universally observed: *in vitro* fermentation of 3’-FL with infant fecal microbiota failed to elevate butyrate concentrations, whereas fermentation of the non-fucosylated HMO precursor lacto-N-biose (LNB) resulted in a significant reduction in butyrate, concomitant with increased lactate and acetate accumulation (Rubio-Del-Campo et al., 2020). Strikingly, microbiome metabolic activity can be profoundly altered even in the absence of shifts in the relative abundance of dominant bacterial genera. In infants born to secretor-positive mothers—but not to secretor-negative mothers—the relative abundances of *Bifidobacterium* and *Streptococcus* exhibit a significant positive correlation and jointly associate with compositional features of the fecal metabolome (Wang et al., 2023). Specifically, in this 2’-FL-rich environment, higher levels of both genera are linked to lower fecal butyrate, propionate, and succinate, and higher levels of lactate and 1,2-PD, suggesting a functional synergy or convergence (Wang et al., 2023). Moreover, infants fed 2’-FL-supplemented formula exhibit significantly reduced plasma concentrations of pro-inflammatory cytokines—including TNF-α—resulting in an immune profile that more closely resembles that of exclusively breastfed infants (Komatsu et al., 2026). Mechanistically, such immunomodulatory effects can be direct: lacto-N-fucopentaose III (LNFP III) interacts with the Toll-like receptor 4 (TLR4)–MD-2–CD14 receptor complex on dendritic cells via its α1,3-fucose moiety—a signaling event that promotes a Th2-skewed immune response (Kononova et al., 2021). This anti-inflammatory effect has been corroborated at the tissue level in humanized murine models: dietary supplementation with 3FN significantly reduced TNF-α protein expression in the colonic mucosa while concurrently upregulating expression of the anti-inflammatory cytokines interleukin-10 (IL-10) and interleukin-13 (IL-13), as well as the pattern recognition receptor Toll-like receptor 2 (TLR2). This anti-inflammatory effect has been corroborated at the tissue level in humanized mice, where dietary 3FN not only reduced levels of the pro-inflammatory cytokine TNF-α in the large intestine but also upregulated the anti-inflammatory cytokines IL-10 and IL-13 and the pattern recognition receptor TLR2 (Rubio-Del-Campo et al., 2021). Broader metabolic shifts are further substantiated by *in vitro* fermentation studies: supplementation with 2’-FL significantly elevates total SCFA concentrations and induces substantial modulation of amino acid, purine, and lipid metabolic pathways (Luo et al., 2026). Notably, the observed increase in *Bifidobacterium* abundance exhibits a statistically significant correlation with alterations in multiple lipid metabolites—highlighting a functionally relevant link between fHMO utilization and infant gut lipid metabolism (Luo et al., 2026); relatedly, systemic effects on circulating lipid profiles have been observed in humanized murine models: supplementation with 3’-FL significantly reduced serum HDL-cholesterol concentrations (Rubio-Del-Campo et al., 2021). The metabolic fate of fHMOs has been traced to specific microbial fermentation end-products; dense longitudinal sampling demonstrates that fucose liberated from fHMOs serves as a primary substrate for formate biosynthesis by *Bifidobacterium* species (Tsukuda et al., 2021). This metabolic activity shows significant inter- and intraspecific variation among bifidobacteria and can involve cross-feeding, where fucose released by one strain is consumed by another, collectively shaping the gut formate pool (Tsukuda et al., 2021).

In addition to their well-established prebiotic and immunomodulatory functions, fHMOs have been increasingly implicated in supporting early neurodevelopment. Notably, longitudinal observational studies report a statistically significant association between infant exposure to 2’-FL and elevated cognitive performance at 24 months of age (Komatsu et al., 2026). Moreover, fHMOs and HMGs have been shown to modulate intestinal epithelial function directly—without requiring microbial mediation. For instance, preclinical studies in piglet models have demonstrated that dietary supplementation with 2’-FL enhances structural and functional maturation of the small intestine, as evidenced by increased villus height and crypt depth, along with upregulated expression of critical nutrient transporters—including SGLT1 and GLUT2 (Komatsu et al., 2026). The piglet is widely regarded as a highly relevant preclinical model for human intestinal development and nutrition, owing to its physiological and anatomical similarities to the human gastrointestinal tract. This relevance is further strengthened by the substantial compositional overlap between porcine and HMOs—particularly in terms of fucosylation patterns and core structural motifs. Although porcine milk exhibits lower structural diversity compared with human milk, its overall oligosaccharide composition is markedly more similar to that of human milk than to bovine milk. Notably, fucosylated oligosaccharides constitute over 9% of the total oligosaccharide pool in porcine milk—compared with only approximately 1% in bovine milk—highlighting a key biochemical distinction relevant to translational nutrition research (Salcedo et al., 2016). This includes human-typical oligosaccharide structures—such as 2’-FL and, more recently, LDFT—which have been identified in porcine milk. Moreover, the developmental trajectory of glycans in porcine milk is distinctive: the relative abundance of fucosylated oligosaccharides increases significantly during the first 2 weeks of lactation, whereas sialylated oligosaccharides concurrently decline (Salcedo et al., 2016). This dietary shift corresponds with the presence of fucose-consuming bacterial taxa in the nursing piglet’s gut, reinforcing the pig as a valuable model for studying the effects of early-life nutrition on gastrointestinal development (Salcedo et al., 2016).

Specific fHMOs confer direct anti-infective activity against enteric pathogens; for example, 2’-FL has been shown to inhibit the cellular invasion of enterotoxigenic *Escherichia coli* (ETEC) *in vitro* using human intestinal epithelial cell models (Komatsu et al., 2026), while higher concentrations of LNnT are associated with a *C. jejuni* reduced risk of *Campylobacter* gastroenteritis (Rubinstein et al., 2025). The protective activity of fHMOs against *Campylobacter* extends beyond LNnT; mechanistic investigations demonstrate that 2’-FL potently inhibits key virulence processes in, including adhesion to and invasion of human intestinal epithelial cells. *In vitro*, treatment of human colonic epithelial HT-29 cells with 2’-FL reduced invasion by *C. jejuni* by approximately 80% and significantly attenuated the secretion of key pro-inflammatory cytokines—namely, interleukin-8 (IL-8) and IL-1β (Yu et al., 2016). These *in vitro* findings were corroborated *in vivo*: oral administration of 2’-FL to a murine model of *C. jejuni* infection resulted in an approximately 80% reduction in cecal bacterial colonization and a 50–70% attenuation of histopathological indicators of intestinal inflammation—including crypt hyperplasia, immune cell infiltration, and epithelial damage (Yu et al., 2016). This protective effect is further augmented by fucosylated glycans present on milk glycoproteins—including secretory immunoglobulin A (sIgA)—whose fucosylated glycotopes function as decoy receptors for enteric pathogens, thereby bridging innate and adaptive immune defense mechanisms (Orczyk-Pawiłowicz and Lis-Kuberka, 2020). The mechanisms underlying fHMO-mediated protection against ARIs appear to operate independently of local respiratory microbiota composition; notably, the reduced ARI incidence observed in infants born to secretor-positive mothers was not correlated with nasopharyngeal microbial community structure—suggesting a primary role for systemic immune modulation or direct pathogen decoy activity (Binia et al., 2021). Indeed, a portion of ingested HMOs is absorbed into the bloodstream and excreted in the urine, where they may act as systemic decoys to prevent sepsis or urinary tract infections (Underwood et al., 2015). This differential fate of HMOs was observed in premature infants, where the class of HMOs containing both fucose and sialic acid appeared to be minimally consumed by the gut microbiota (as they increased in feces) while also being absorbed from the gut (as they increased in urine) (Underwood et al., 2017). The differential urinary versus fecal abundance of specific HMOs holds promise as a non-invasive biomarker for assessing intestinal barrier integrity in infants (Underwood et al., 2015). In a fascinating twist, the capacity to produce fHMOs may not be exclusive to the host; analysis of the gut virome has revealed that prophages within bacterial genomes can carry a functional α-1,2-fucosyltransferase (*futC*) gene capable of directing 2’-FL biosynthesis, which was shown to alleviate colitis in a mouse model (Gao et al., 2025).

### 3.3 Microbial fucose metabolism

**FIGURE 7:**
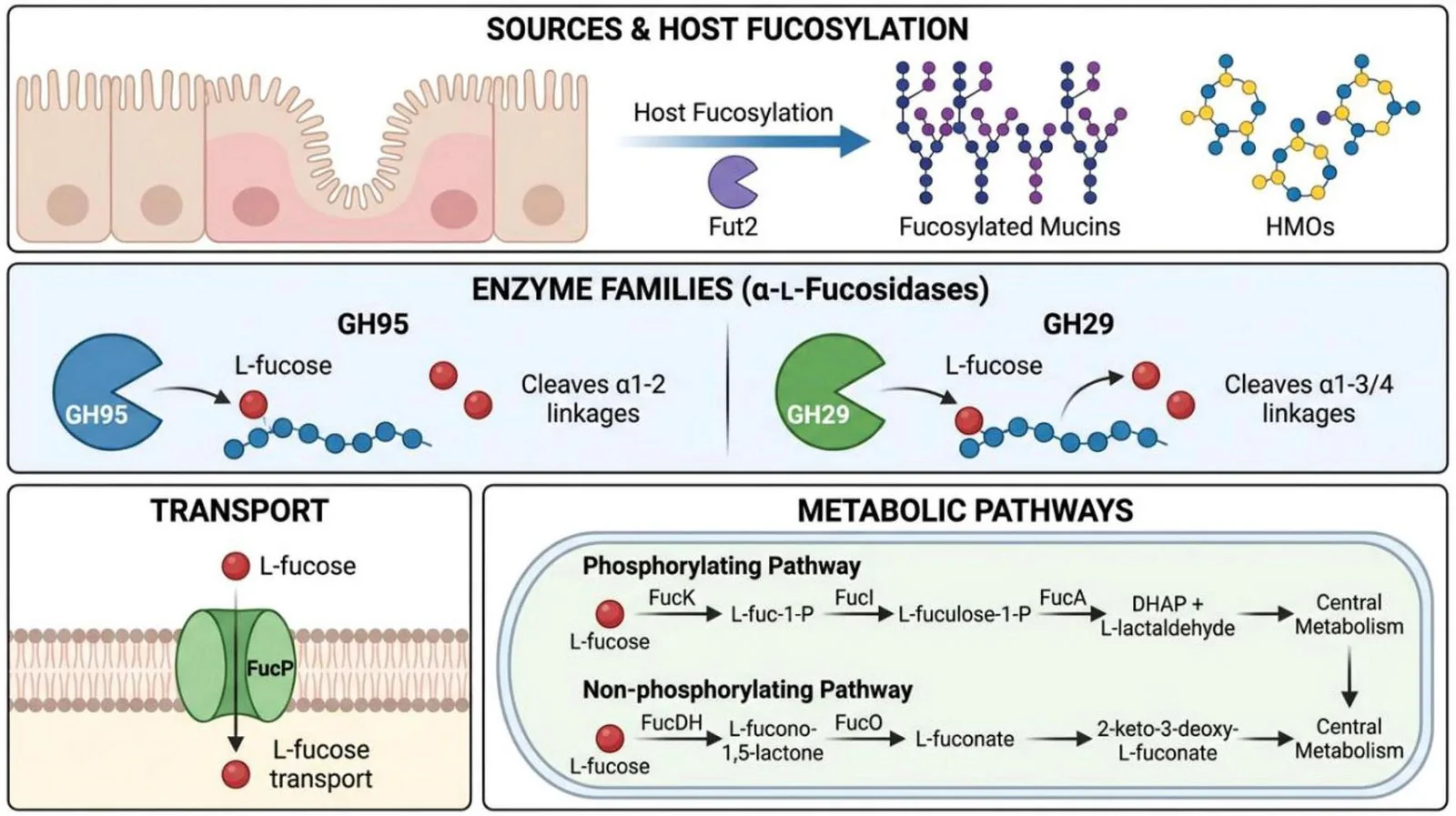
Microbial L-fucose utilization, conferred by genes for transporters and fucosidases, provides a competitive advantage (Kim et al., 2023). The key enzymes are α-L-fucosidases from glycosyl hydrolase (GH) families GH29 and GH95; the role of GH151 is debated due to poor activity on fucosylated substrates (Curiel et al., 2021). These enzymes have distinct specificities: GH95 enzymes use an inverting mechanism on α1,2-bonds, while GH29 enzymes use a retaining mechanism. GH29 is further divided, with subfamily GH29-A showing broad specificity and GH29-B targeting α1,3/α1,4 linkages, allowing different bacteria to access specific fucosylated glycans on mucins or HMOs (Curiel et al., 2021; Wu et al., 2021).

Keystone gut commensals employ distinct fucose utilization strategies (Figure 7 and Table 3). *Bifidobacterium infantis* uses a “selfish” intracellular strategy, internalizing intact fHMOs and cleaving fucose with intracellular GH29 and GH95 fucosidases (Sakanaka et al., 2019; Curiel et al., 2021). In contrast, *Bifidobacterium bifidum* acts as a public goods producer, using extracellular fucosidases to release free fucose for cross-feeding (Curiel et al., 2021). The mucin-degrader *A. muciniphila* also targets fucosylated mucins (Trastoy et al., 2020). *Ruminococcus gnavus* strains exhibit strain-specific metabolic adaptations: the E1 strain—despite lacking sialidase activity—encodes a specialized GH29 α-fucosidase capable of directly hydrolyzing α1,3/4-linked fucose residues from sialylated glycan epitopes, including sialyl Lewis X (sLeX), thereby circumventing the requirement for prior desialylation (Wu et al., 2021).

**TABLE 3:** Microbial fucose metabolism: key enzymes and bacterial strategies.

Name	Content/function	Citation (PMID)
GH29 α-L-fucosidase	Key enzyme with a retaining mechanism. Subfamily GH29-A has broad specificity; GH29-B targets α1,3/α1,4 linkages.	(Curiel et al., 2021; Wu et al., 2021)
GH95 α-L-fucosidase	Key enzyme with an inverting mechanism, specific for α1,2-bonds.	(Curiel et al., 2021)
GH151 α-L-fucosidase	The role in fucose metabolism is debated due to poor activity on fucosylated substrates.	(Curiel et al., 2021)
*Bifidobacterium longum* subsp. *infantis*	Employs a “selfish” intracellular strategy, internalizing intact fucosylated HMOs before cleaving fucose.	(Sakanaka et al., 2019; Curiel et al., 2021)
*Bifidobacterium bifidum*	Acts as a “public goods” producer, using extracellular fucosidases to release free fucose for cross-feeding.	(Curiel et al., 2021)
*A. muciniphila*	Mucin-degrader that targets and utilizes fucosylated mucins.	(Trastoy et al., 2020)
*Ruminococcus gnavus* (E1 strain)	Possesses a unique GH29 fucosidase that directly cleaves fucose from sialylated structures (e.g., sLeX).	(Wu et al., 2021)
*Bacteroides* spp.	Significant consumers of HMOs in the infant gut (e.g., *B. dorei*); the fucose-metabolizing activity of *B. thetaiotaomicron* is mechanistically coupled to its induction of host epithelial fucosylation.	(Bry et al., 1996; Kijner et al., 2022)
*Lactobacillus* spp.	Beneficial commensal genus whose proliferation is supported by the fermentation of fucosylated HMOs, such as 2’-FL.	(Luo et al., 2026)

GH, glycoside hydrolase (family); HMO, human milk oligosaccharide; 2’-FL, 2’-fucosyllactose; sLeX, sialyl-Lewis X; subsp., subspecies; spp., species (plural); PMID, PubMed identifier. α1, 2-, α1,3- and α1,4- denote fucose glycosidic linkage positions.

L-Fucose catabolism proceeds via the 1,2-PD pathway, yielding 1,2-PD as an intermediate that is subsequently converted to propionate. In addition to propionate, this metabolic route generates immunomodulatory metabolites—including lactate, acetate, and formate—as well as other SCFAs, thereby facilitating syntrophic cross-feeding among gut commensals (Curiel et al., 2021; Kim et al., 2023). Critically, both host-derived fucosylation—mediated by the fucosyltransferase FUT2—and bacterial surface fucosylation are indispensable for maintaining intestinal immune homeostasis and constraining inflammation, indicating a bidirectional, glycan-mediated dialogue between host and microbiota (Lei et al., 2025).

### 3.4 Syntrophy and cross-feeding driven by fucose metabolism

**FIGURE 8:**
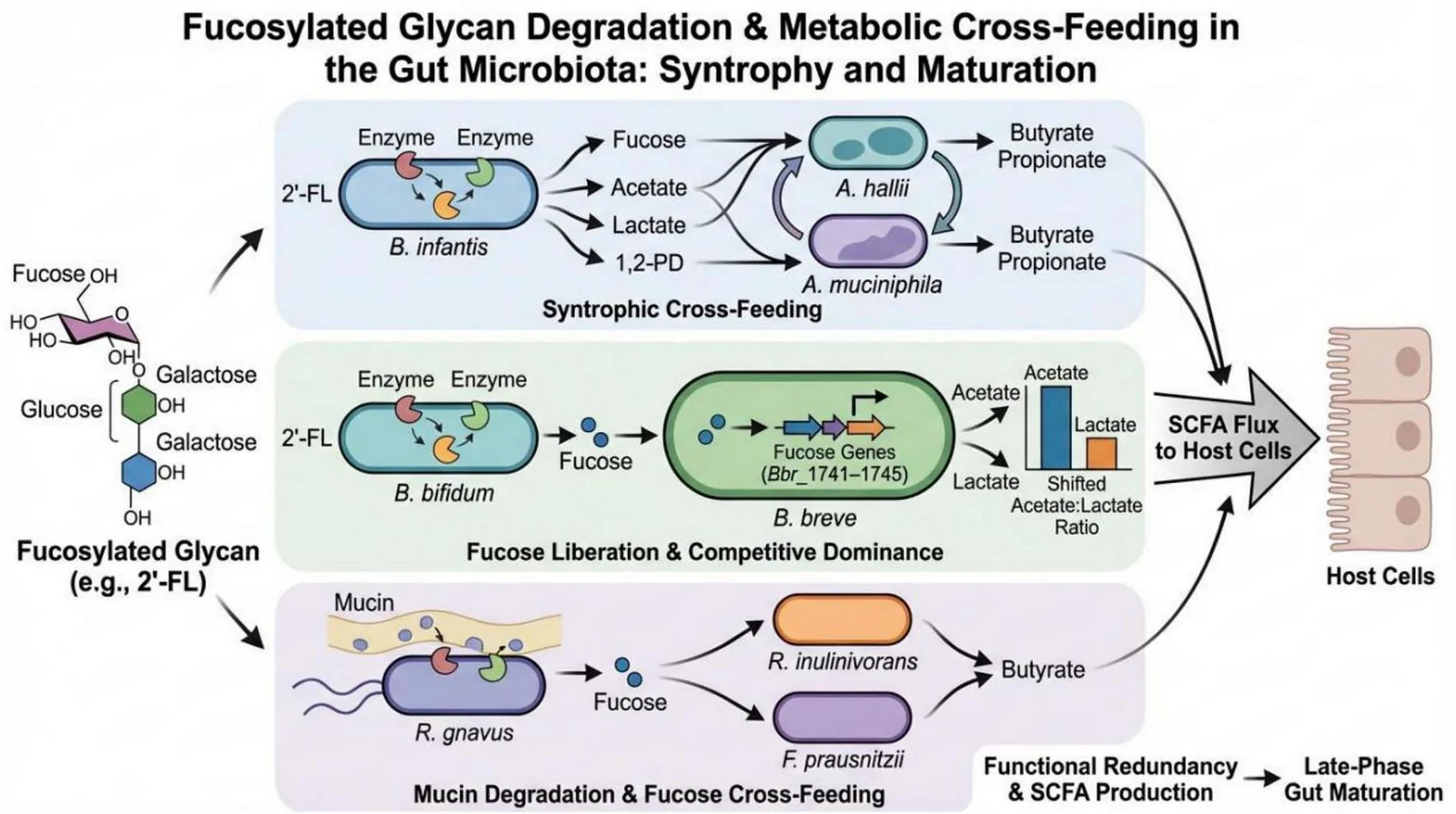
Degradation of fucosylated glycans (e.g., HMOs, mucins) initiates a metabolic cascade where primary degraders release byproducts that support secondary consumers, particularly butyrate producers, through syntrophy and cross-feeding (Berkhout et al., 2022). For example, *Bifidobacterium infantis* metabolizes fHMOs like 2’-fucosyllactose (2’-FL), releasing fucose, acetate, lactate, and 1,2-PD. The butyrate producer *A. hallii* consumes these byproducts, converting acetate and lactate to butyrate and metabolizing 1,2-PD to propionate. This process can require a syntrophic partner like *A. muciniphila* as a hydrogen sink (Berkhout et al., 2022). This syntrophy and the resulting SCFA flux are specific to the fHMO structure and *Bifidobacterium infantis* strain (Dedon et al., 2023).

*Bifidobacterium breve* functions as a secondary consumer, feeding on fucose released by primary degraders such as *Bifidobacterium bifidum* (Figure 8 and Table 4). In a gnotobiotic mouse model, wild-type *Bifidobacterium breve* co-colonized with *Bifidobacterium bifidum* reached levels 100-fold higher than its partner, an advantage lost by a mutant unable to metabolize fucose. *In vivo* transcriptomics confirmed *Bifidobacterium breve* upregulated fucose metabolism genes (Bbr_1741–1745) (O’Connell Motherway et al., 2018). Similarly, in an *in vitro* chemostat co-culture system supplemented with 2’-FL, *Bifidobacterium breve* emerged as the dominant population, whereas transcriptomic analysis revealed that the fucose-donating partner, *Bifidobacterium bifidum*, coordinately upregulated genes encoding carbohydrate transporters and the α-L-fucosidase responsible for liberating fucose from 2’-FL (Centanni et al., 2019).

**TABLE 4:** Bacterial roles in fucose-driven syntrophy and cross-feeding.

Name	Content/function	Citation (PMID)
*Bifidobacterium infantis*	Primary degrader of fHMOs; releases fucose, acetate, lactate, and 1,2-PD for cross-feeding.	(Berkhout et al., 2022; Dedon et al., 2023)
*Anaerobutyricum hallii* (*A. hallii*)	Secondary consumer; utilizes acetate and lactate to produce butyrate, and metabolizes 1,2-PD to propionate.	(Berkhout et al., 2022)
*A. muciniphila*	Syntrophic partner; acts as a hydrogen sink, facilitating metabolism by partners like *A. hallii*.	(Berkhout et al., 2022)
*Bifidobacterium bifidum*	Primary degrader; liberates fucose from 2’-FL, which is then consumed by secondary feeders like *B. breve*.	(O’Connell Motherway et al., 2018; Centanni et al., 2019)
*Bifidobacterium breve*	Secondary consumer; cross-feeds on fucose liberated by primary degraders, leading to its dominance in co-culture.	(O’Connell Motherway et al., 2018; Centanni et al., 2019)
*Ruminococcus gnavus*	Primary degrader of mucin; releases fucose that supports non-degrading species.	(Berkhout et al., 2022)
*Roseburia inulinivorans*	Secondary consumer; utilizes fucose (producing lactate/acetate) and 1,2-PD (producing propionate) from primary degraders.	(Berkhout et al., 2022)
*Faecalibacterium prausnitzii*	Secondary consumer; utilizes fucose released by primary mucin degraders like *R. gnavus*.	(Berkhout et al., 2022)
*Clostridiales* species	Secondary consumers in the maturing infant gut; cross-feed on acetate from primary fermenters to produce butyrate and propionate.	(Tsukuda et al., 2021)
*Lactobacillus casei*	Internalizes fucosylated HMOs (e.g., Fuc-α1,3-GlcNAc) and metabolizes the N-acetylglucosamine moiety intracellularly, excreting free fucose that becomes available for cross-feeding by other members of the gut community.	(Ibiza et al., 2016)

fHMO, fucosylated human milk oligosaccharide; 2’-FL, 2’-fucosyllactose; 1,2-PD, 1,2-propanediol; Fuc-α1,3-GlcNAc, α1,3-fucosylated N-acetylglucosamine; PMID, PubMed identifier.

This metabolic interaction extends to mucin degradation, in which *Ruminococcus gnavus* liberates fucose from mucin glycans—thereby providing a key carbon source for non-mucinolytic commensals such as *Roseburia inulinivorans* and *Faecalibacterium prausnitzii*. *R. inulinivorans* subsequently ferments fucose to lactate and acetate and converts 1,2-PD to propionate, thereby establishing a multi-step, cross-feeding metabolic partnership (Berkhout et al., 2022).

In complex modeled microbiomes, fHMO structure determined community composition but not net SCFA production due to functional redundancy (Dedon et al., 2023). Conversely, in a simple *Bifidobacterium bifidum*/ *Bifidobacterium breve* co-culture, the acetate-to-lactate ratio shifted from ∼2:1 to ∼8:1 (Centanni et al., 2019). L-fucose metabolism generates a significant SCFA flux for host cells (Kim et al., 2023). Late-phase maturation of the infant gut microbiota is characterized by increased abundances of propionate and butyrate, which correlate with the expansion of acetate-consuming *Clostridiales* species—particularly those engaged in cross-feeding interactions with primary saccharolytic fermenters (Tsukuda et al., 2021).

## 4 The role of fucosylation in intestinal health and disease pathogenesis

**FIGURE 9:**
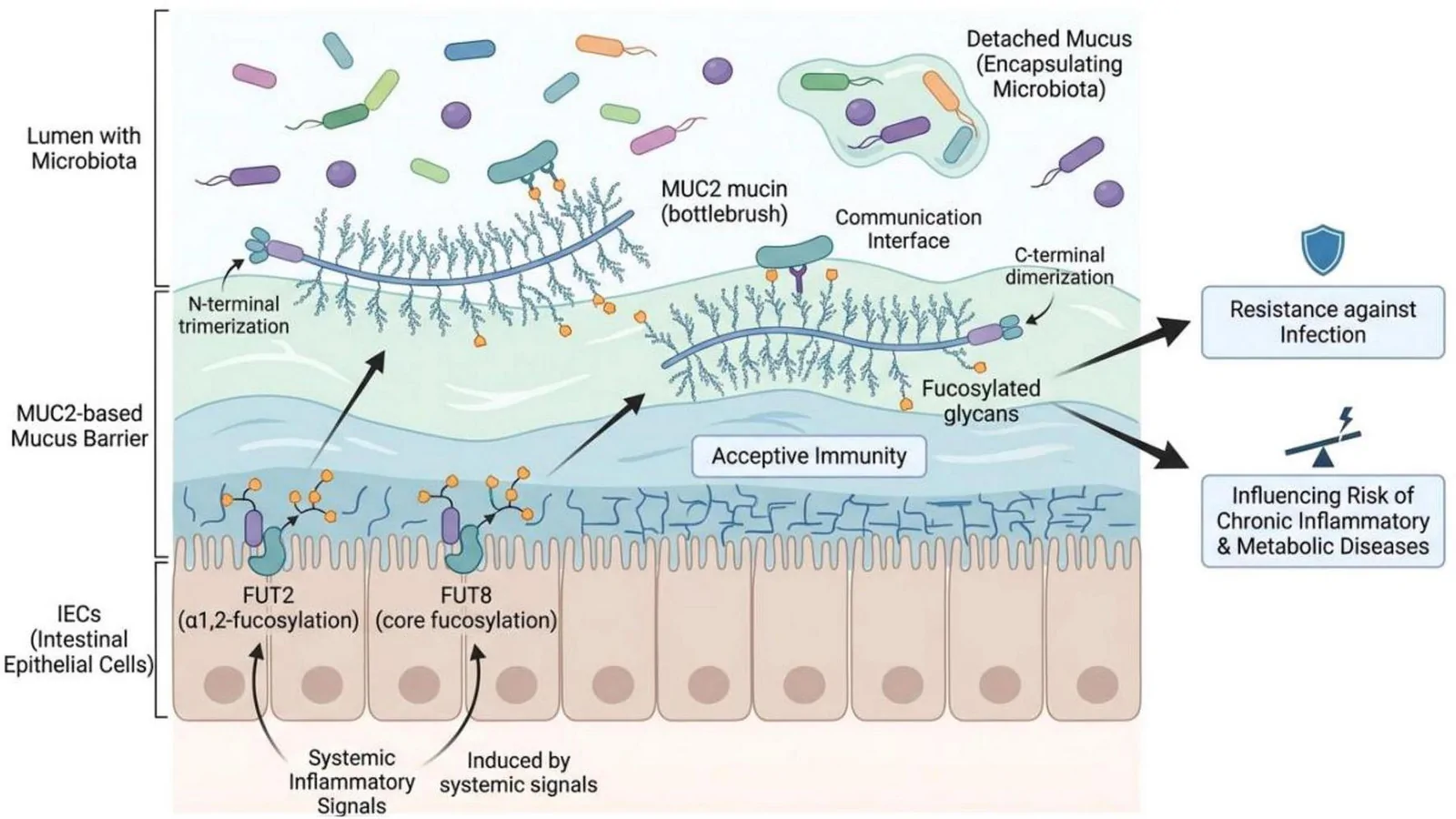
Schematic overview of the intestinal MUC2-based mucus barrier. The intestinal lumen contains commensal microbiota, while overlying goblet cells within the intestinal epithelium secrete MUC2 mucin that assembles into a cohesive “bottlebrush” network through N-terminal trimerization and C-terminal dimerization. Epithelial expression of *FUT2* (mediating α1,2-fucosylation) and *FUT8* (mediating core fucosylation) generates fucosylated glycans on MUC2, a process induced by systemic inflammatory cues. This barrier provides physical protection by shedding detached mucus layers that encapsulate microbes to limit pathogen invasion (resistance against infection), while also establishing a communication interface with the microbiota that supports acceptive immunity and modulates the risk of chronic inflammatory and metabolic diseases.

Intestinal fucosylation orchestrates key aspects of mucosal physiology—barrier integrity, host–microbiota crosstalk, and immune homeostasis—and its dysregulation is a recurring determinant of intestinal disease pathogenesis (Li Y. et al., 2025) (Figure 9 and Table 5). The host immune system must not only protect against pathogens but also establish homeostatic relationships with the vast community of symbiotic microorganisms—a concept termed “acceptive immunity” (Li Y. et al., 2025). Fucosylation is a key molecular language in this dialogue. The core components of this barrier are mucins—particularly the secreted, gel-forming MUC2—which polymerize via N-terminal trimerization and C-terminal dimerization to form an extensive network structure. This network acquires its characteristic “bottlebrush-like” appearance and viscosity from dense O-glycosylation along its proline-, threonine-, and serine-rich protein core (Okumura and Takeda, 2024; Cheng et al., 2025). The resulting fucosylated glycans on IECs and within these mucin structures serve as both a physical shield and a dynamic communication interface, with specific glycan modifications acting as a decisive determinant of mucin function (Cheng et al., 2025).

**TABLE 5:** Roles of intestinal fucosylation in health and disease: mechanisms and outcomes.

Context/disease	Role of fucosylation: mechanisms and outcomes	Citation (PMID)
Colonization resistance (physiological)	Fucosylated glycans on IECs and mucins provide adhesion sites and nutrients for commensals, shaping a community that outcompetes invaders; direct fucose competition mediates pathogen exclusion (e.g., *Dubosiella newyorkensis* depletes fucose required by MRSA).	(Cheng et al., 2025; Lei et al., 2025; Li and Guo, 2026)
Anti-infective defense	Soluble fucosylated HMOs (e.g., 2’-FL, LNFP I) act as decoy receptors that block pathogen adhesion (*C. jejuni*, EV71, *H. pylori*); 2’-FL attenuates *C. jejuni* invasion of epithelial cells by up to 80% and suppresses IL-8/IL-1β release.	(Ruiz-Palacios et al., 2003; Yu et al., 2016; Gao et al., 2022; Komatsu et al., 2026)
Immune homeostasis and tolerance	Fucose-containing Lewis x motifs on commensals engage DC-SIGN on dendritic cells, promoting Th2/Treg tolerogenic programs; bacterial surface fucosylation enables IgA recognition, and dietary fucose enhances IgA-mediated protection against *Salmonella*.	(Tytgat et al., 2016; Kononova et al., 2021; Lei et al., 2025)
Inducible fucosylation (IL-22–FUT2 Axis)	Systemic infection or inflammation triggers IL-23/IL-22 signaling that upregulates epithelial *Fut2*, supporting commensals, maintaining colonization resistance during colitis [e.g., against *Citrobacter rodentium* (*C. rodentium*) and *Enterococcus faecalis* (*E. faecalis*)] and mitigating colitis severity; pathogen-induced Wnt/β-catenin signaling also upregulates FUT8-mediated core fucosylation against *Salmonella*.	(Pham et al., 2014; Pickard et al., 2014; Hao et al., 2020)
Inflammatory bowel disease (IBD)	*FUT2* loss-of-function (non-secretor status) is a well-established Crohn’s disease risk factor; reduced α1,2-fucosylation drives dysbiosis (depletion of anti-inflammatory taxa, expansion of *E. coli*), weakens the mucus barrier, and lowers bacterial surface fucosylation, enabling evasion of IgA surveillance and aggravating inflammation.	(Rausch et al., 2011; Tang et al., 2021; Hu et al., 2022; Sharma et al., 2025)
Obesity and metabolic dysfunction	Fut2-mediated fucosylation is context-dependent: endogenous fucosylation promotes high-fat-diet-induced obesity and steatohepatitis via microbiota-driven bile acid remodeling and intestinal FXR signaling (*Fut2*-deficient mice are protected), whereas exogenous 2’-FL protects by thickening the mucus layer, enriching *Akkermansia*, and increasing GLP-1/PYY secretion.	(Pickard et al., 2014; Zhou R. et al., 2021; Paone et al., 2024)
*Alcohol-related liver disease (ALD)*	Protective role: α1,2-fucosylation is downregulated in patients; intestinal *Fut2* deficiency exacerbates ethanol-induced liver injury in a microbiota-dependent manner, while 2’-FL supplementation ameliorates pathology by limiting cytolysin-producing *E. faecalis* and preserving hepatic NAD^+^ homeostasis (gut–liver axis).	(Zhou et al., 2020; Chen L. et al., 2025)
Colorectal cancer (CRC)	GDP-fucose synthesis defects (*GMDS* loss in 6-13% of CRCs, enriched in right-sided tumors) impair mucin fucosylation, abrogate Notch/HES1 signaling, and drive inflammation-associated carcinogenesis; under-fucosylated MUC2 compromises the barrier, while sialyl-Lewis antigens built on fucosylated scaffolds engage E-selectin to promote metastasis.	(Nakagoe et al., 2000; Pinho and Reis, 2015; Wang et al., 2017; Li Y. et al., 2025)

IEC, intestinal epithelial cell; MRSA, methicillin-resistant *Staphylococcus aureus*; HMO, human milk oligosaccharide; 2’-FL, 2’-fucosyllactose; LNFP I, lacto-N-fucopentaose I; DC-SIGN, dendritic cell-specific ICAM-3-grabbing non-integrin; Th2, T helper 2 cell; Treg, regulatory T cell; IgA, immunoglobulin A; IL, interleukin; FUT, fucosyltransferase; EV71, enterovirus 71; FXR, farnesoid X receptor; GLP-1, glucagon-like peptide-1; PYY, peptide YY; NAD+, nicotinamide adenine dinucleotide; GDP, guanosine diphosphate; GMDS, GDP-mannose-4,6-dehydratase; MUC2, mucin 2; PMID, PubMed identifier. α1,2- denotes the fucose glycosidic linkage position.

This physical shield is not merely a static, two-layered gel but a dynamic system: recent models propose that detached mucus in the colon actively encapsulates luminal contents, thereby organizing the microbiota into discrete communities during gut transit (Okumura and Takeda, 2024). Moreover, fucosylation is not static; it can be rapidly and dynamically induced in the small intestine in response to systemic inflammatory signals—serving as an adaptive host mechanism that supports the microbiota during periods of stress (Pickard et al., 2014). Consequently, host fucosylation status is intricately linked to a spectrum of physiological and pathological outcomes, ranging from resistance to infection to modulation of the risk for chronic inflammatory and metabolic diseases.

### 4.1 Colonization resistance and infectious disease

The fucosylated glycocalyx of the intestinal epithelium is a key determinant in establishing a healthy, symbiotic microbiota and defending against pathogens. This relationship reflects deep coevolution, as the establishment of the fucosylated landscape itself depends on signals from the resident microbiota. Classic gnotobiotic studies demonstrated this host specificity, showing that colonization of germ-free mice with mouse-derived microbiota—but not with rat- or human-derived microbiota—was required to induce synthesis of the fucosylated glycolipid fucosyl-asialo-GM1 on the intestinal epithelial surface (Okada et al., 1994). These host-derived glycans provide specific adhesion sites and a crucial nutrient source for commensal bacteria, thereby shaping a microbial community capable of outcompeting potential invaders—a concept known as colonization resistance (Lei et al., 2025). Indeed, specific components of mucin glycan chains, including fucose, serve as key nutrients that support the growth of beneficial microbes while inhibiting the proliferation of harmful ones (Cheng et al., 2025). This competition for fucose constitutes a direct mechanism of pathogen exclusion. For instance, the commensal bacterium *Dubosiella newyorkensis* has been shown to mediate colonization resistance against methicillin-resistant *Staphylococcus aureus* (MRSA) by actively depleting fucose—a carbon source required by MRSA for growth and pathogenicity (Li and Guo, 2026).

Beyond serving as a niche for commensals, fucosylated glycans play a direct role in anti-infective defense. Many enteric pathogens have evolved to recognize and bind specific fucosylated structures on the host cell surface as a first step in infection establishment. A classic example is *C. jejuni*—a leading cause of bacterial diarrhea—that binds with high avidity to the intestinal H1 antigen (Fucα1,2Galβ1,4GlcNAc) (Ruiz-Palacios et al., 2003). This host–pathogen interaction can be effectively thwarted by a sophisticated host defense mechanism: the secretion of soluble fucosylated oligosaccharides. HMOs, particularly fucosylated structures such as 2’-FL, are abundant in breast milk and function as soluble decoy receptors. These free glycans mimic epithelial surface binding sites, competitively inhibiting pathogen adhesion and preventing colonization (Ruiz-Palacios et al., 2003; Komatsu et al., 2026).

*In vitro* studies have demonstrated that 2’-FL attenuates *C. jejuni* invasion of epithelial cells by up to 80% and suppresses the subsequent inflammatory response by inhibiting the release of pro-inflammatory mediators such as IL-8 and IL-1β (Yu et al., 2016). This principle extends to viral pathogens; for instance, lacto-N-fucopentaose I (LNFP I), another prominent fucosylated HMO, inhibits Enterovirus 71 (EV71)—a primary cause of hand, foot, and mouth disease—by binding directly to the viral capsid protein VP1, thereby blocking viral adsorption to host cells and protecting them from virus-induced apoptosis (Gao et al., 2022). More broadly, fucosylated glycans and histo-blood group antigens (HBGAs) are exploited by major enteric viruses during host recognition and attachment: rotaviruses and noroviruses bind HBGA glycans on intestinal epithelial cells, and host secretor (*FUT2*) status is a key determinant of susceptibility to these infections (Peña-Gil et al., 2021). This protective mechanism extends beyond the intestine, as fucosylated HMOs can also prevent *Helicobacter pylori* infection in the stomach via a similar decoy mechanism (Komatsu et al., 2026), consistent with the bacterium’s reliance on α1,2-fucosylated glycans in the gastric mucosa for adhesion and colonization (Sitkin et al., 2022). This interaction further extends to the adaptive immune system, where both host-derived and bacterial surface fucosylation are critical for immunoglobulin A (IgA) recognition of the microbiota—a key process in immune surveillance and homeostasis. Dietary fucose enhances this IgA response, conferring protection against pathogens such as *Salmonella* by preventing systemic dissemination (Lei et al., 2025).

A critical mechanism underlying immune tolerance to the gut microbiota involves the C-type lectin receptor DC-SIGN (Dendritic Cell–Specific Intercellular Adhesion Molecule-3–Grabbing Non-integrin), expressed on immature dendritic cells, which discriminates between structurally distinct microbial carbohydrate motifs to direct T-cell polarization (Kononova et al., 2021). Specifically, engagement with mannose-rich pathogen-associated ligands typically drives protective Th1 responses, whereas binding to fucose-containing commensal-associated motifs such as Lewis x (Lex) promotes a tolerogenic program characterized by Th2 skewing and robust regulatory T cell (Treg) induction. This signaling pathway suppresses pro-inflammatory cytokines (e.g., IL-12) while enhancing anti-inflammatory IL-10 production. Such DC-SIGN–mediated immunomodulation is co-opted by both pathogens and commensal microbes through molecular mimicry to shape host immunity: for example, Lex antigens from *Helicobacter pylori* lipopolysaccharide (LPS) or the helminth Schistosoma mansoni soluble egg antigen engage DC-SIGN to drive Th2 polarization and suppress protective Th1 immunity; meanwhile, the identical Lex epitope on commensal *Bacteroides* species functions as a bona fide microbe-associated molecular pattern (MAMP) that sustains intestinal tolerance. This paradigm is further reinforced by the probiotic *Lactobacillus rhamnosus* GG, whose fucosylated SpaCBA pili—post-translationally glycosylated with fucose and mannose—specifically bind DC-SIGN on dendritic cells, an interaction indispensable for calibrating cytokine output and reinforcing mucosal immune homeost (Tytgat et al., 2016).

Intestinal fucosylation represents a dynamic, inducible host defense mechanism beyond its constitutive role, responding to systemic infection, inflammation, and neural cues to modulate microbial symbiosis and enhance fitness. At its core, systemic exposure to microbial products like LPS triggers dendritic cell TLR sensing, leading to IL-23/IL-22 production that stimulates IECs to upregulate *Fut2* and α1,2-fucosylate the small intestine—a process critical for supporting commensals, maintaining colonization resistance (e.g., against *Citrobacter rodentium* or *Enterococcus faecalis* during colitis), and improving infection recovery—though it does not reduce pathogen load, it mitigates colitis severity, an effect lost in the absence of gut microbiota (Pham et al., 2014; Pickard et al., 2014). Beyond FUT2, core fucosylation catalyzed by FUT8 serves as another inducible defense: *Fut8*^+^/^–^ mice exhibit heightened susceptibility to *Salmonella Typhi*, linked to baseline reductions in beneficial *Lactobacillus*, while pathogen-induced Wnt/β-catenin signaling upregulates *FUT8* to remodel the mucosa, promoting fucose-utilizing commensals (*Lactobacillus*, *A. muciniphila*) that antagonize pathogens (Hao et al., 2020).

Notably, the IL-22–FUT2 axis shows context-dependent specificity: while central to bacterial defense, its antiviral role against rotavirus is fucosylation-independent—IL-22 blocks replication in microbiota-depleted mice equally in wild-type and *Fut2*-deficient models, indicating distinct effector mechanisms for viruses (Schnepf et al., 2021). This inducible fucosylation extends to neural regulation via the gut-brain axis: electroacupuncture activates the vagus nerve to upregulate *Fut2*, enhancing barrier integrity (MUC2^+^ goblet cells and tight junction proteins) and reshaping microbiota (enriching *Lactobacillales*), with neuroprotective effects transferable via fecal microbiota transplant only when the donor’s vagus nerve is intact (Zhao et al., 2026).

Host fucosylation also shapes probiotic engraftment: *Fut2* genotype determines persistence of fucose-utilizing probiotics (e.g., *Bifidobacterium infantis*) post-antibiotics, with secretor-positive mice supporting colonization while non-secretor mice cannot—explaining interindividual variability in probiotic efficacy (Wang et al., 2024). Clinically, this principle informs malnutrition therapy: a “repaired” microbiome (enriched in *Prevotella copri*) from dietary intervention induces duodenal/jejunal goblet cell *Fut2* upregulation, increasing α1,2-fucosylation to create a symbiotic niche that promotes growth—illustrating a feedback loop where diet–microbiome–host glycosylation interactions restore health (Wang et al., 2025). Collectively, these findings highlight fucosylation as a versatile, host-driven strategy to dynamically tune the gut ecosystem across infection, neural stress, and therapeutic contexts.

### 4.2 Inflammatory bowel disease

IBD exhibits a complex genetic architecture, with loss-of-function polymorphisms in the *FUT2* gene representing one of the most significant and well-established risk factors for Crohn’s disease (CD) (Rausch et al., 2011; Hu et al., 2022). Individuals homozygous for non-functional alleles (e.g., G428A in Caucasians, A385T in Asians)—termed “non-secretors”—are unable to perform α1,2-fucosylation on mucosal surfaces and in secretions, a single genetic trait that increases susceptibility to pathogens (e.g., *E. coli*, *Neisseria meningitidis*, *Candida albicans*) (Sitkin et al., 2022), compromises intestinal homeostasis, elevates chronic inflammation risk, and may influence disease localization (e.g., favoring colonic CD) (Hu et al., 2022). This genetic link also explains the inverse epidemiological association between *Helicobacter pylori* infection and IBD risk: the *FUT2* secretor genotype is a shared underlying factor, independently increasing *H. pylori* susceptibility (via fucosylated gastric mucosal adhesion sites) while protecting against IBD (Sitkin et al., 2022). Mechanistically, *H. pylori* uses fucosylated Lewis antigens to bind DC-SIGN on dendritic cells, promoting a tolerogenic Th2 response over pro-inflammatory Th1 immunity (Kononova et al., 2021). Conversely, the non-secretor phenotype (an IBD risk factor) protects against *H. pylori* colonization (Sitkin et al., 2022). Non-secretors may further face compounded susceptibility due to an inability to mount protective, inducible fucosylation responses during systemic stress—a pathway that supports microbial homeostasis and inflammation tolerance (Pickard et al., 2014). This inducible pathway is driven by cytokines like IL-22, which signals through its epithelial receptor to upregulate *Fut2*; mice lacking the IL-22 receptor are highly susceptible to chemical-induced colitis, linked to uncontrolled expansion of opportunistic pathogens (e.g., *Enterococcus faecalis*) in the absence of fucosylation-supported commensal barriers (Pham et al., 2014). While the *FUT2*–CD association is strong, its role in ulcerative colitis (UC) is ambiguous, acting as a risk factor in some populations (e.g., Han Chinese) but protective in others (e.g., Finnish), underscoring the complex interplay of genetics and environmental factors (Hu et al., 2022). This population heterogeneity likely stems from unmeasured confounders: first, *FUT2* genotype primarily shapes mucosa-associated microbiota, while most cohort studies profile fecal microbiota (which weakly correlates with mucosal glycan-driven niches); second, α1,2-fucosylation interacts with concurrent mucin modifications (e.g., sialylation, sulfation) that vary by diet and geography, obscuring fucosylation-specific effects.

The absence of α1,2-fucosylation profoundly disrupts the gut microbiota. In both CD and UC, *FUT2* expression and α1,2-fucosylation are significantly downregulated in colonic mucosa—reducing fucose content in mucin glycans (Tang et al., 2021; Wang et al., 2022). This impairs commensal adhesion while favoring pathogen attachment, directly fueling inflammation-driving dysbiosis. Distinguishing fucosylation-specific effects from concurrent sialylation changes remains challenging, as the two modifications are often inversely correlated in IBD. Emerging solutions include glycosyltransferase-specific conditional knockouts and mass spectrometry-based glycomics with isotopic tracer labeling of sugar nucleotide fluxes. In a primate model of idiopathic chronic diarrhea (ICD) with UC-like features, fecal metatranscriptomics during active disease revealed upregulated mucin-degrading enzymes and fucose utilization pathways. Host-derived fucosylated mucins thus serve as dual resources for opportunistic pathogens (e.g., *Campylobacter*), which exploit them as carbon source and adhesion platform—and enhance colonization by upregulating adhesion genes (Westreich et al., 2019). Fucosylated glycans are preferred nutrients for beneficial commensals; their loss in non-secretors triggers hallmark IBD dysbiosis: reduced diversity and richness, depletion of anti-inflammatory taxa, and expansion of pro-inflammatory bacteria like *E. coli* (Hu et al., 2022; Sharma et al., 2025). The *FUT2* genotype is a dominant determinant of microbiota composition, explaining substantial inter-individual variation (Rausch et al., 2011). Non-secretors show elevated Bacteroidetes and a lowered Firmicutes/Bacteroidetes ratio—a key IBD signature (Rausch et al., 2011; Sharma et al., 2025). This host–microbe crosstalk is bidirectional: in a cystic fibrosis mouse model (characterized by chronic inflammation and dysbiosis), a Firmicutes-rich/Bacteroidetes-poor microbiota drives ileal upregulation of *Fut2* and *B4galt1*—glycosyltransferases essential for mucosal glycan remodeling (Ikpa et al., 2020). Critically, CD-associated loss of microbial phylogenetic diversity is shaped both by disease activity and *FUT2* genotype. Even healthy non-secretors harbor microbiota more similar to CD patients than to healthy secretors—indicating that the non-secretor genotype intrinsically predisposes the gut to disease-linked dysbiosis (Rausch et al., 2011). Yet mechanisms remain contested: some studies report higher butyrate in non-secretors, suggesting that loss of fucosylated niches may enable overgrowth of inflammatory microbes (Hu et al., 2022). Supporting therapeutic potential, oral administration of the butyrate-producing *F. prausnitzii* UT1 strain reshaped the microbiota in mice—enriching *Lactobacillus* and *Bifidobacterium* while suppressing mucin degraders (e.g., *Ruminococcus gnavus*) and their CAZymes—thereby correcting dysbiosis and protecting mucosal barrier integrity (Geng et al., 2025).

This dysbiosis involves both compositional and functional changes—including altered bacterial surface properties. Host fucosylation directly regulates commensal bacteria’s fucosylation pathways: *Fut2*-deficient mice show markedly reduced bacterial surface fucosylation (Lei et al., 2025). Clinically, IBD patients similarly exhibit decreased bacterial surface fucosylation, correlating with worse intestinal inflammation (Lei et al., 2025). Critically, bacterial surface fucosylation—not just host-derived—is essential for gut immune homeostasis. Microbiota from *Fut2*-deficient mice or low-fucosylating strains like *Bacteroides fragilis* fail to sustain intestinal IgA responses and increase colitis susceptibility in recipient mice (Lei et al., 2025). Mechanistically, surface fucosylation enables IgA binding; its loss allows bacteria to evade IgA surveillance and drive inflammation (Lei et al., 2025). Beyond host and bacterial contributions, the microbiome itself produces fucosylated molecules—e.g., a prophage-encoded α-1,2-fucosyltransferase (futC) is depleted in IBD patients’ viromes (Gao et al., 2025). This enzyme synthesizes 2’-FL; exogenous 2’-FL ameliorates colitis in mice by enhancing mucosal barrier function, increasing IgA secretion, and promoting intraepithelial CD4^+^CD8αα^+^ T-cell development—in a microbiota-dependent manner (Gao et al., 2025).

Fucosylation deficiency directly impairs the integrity of the mucus barrier. Mucin glycosylation endows mucus with physicochemical properties to resist enzymatic degradation and form a physical barrier to separate the microbiota from the epithelium (Bergstrom and Xia, 2013; Li Y. et al., 2025), especially crucial in the stratified mucus structure of the colon (Bergstrom and Xia, 2013). Defects in core O-glycan synthesis also disrupt barrier function: mice with epithelium-specific knockout of core 1 O-glycan synthase *C1GalT1* develop severe spontaneous colitis, recapitulating the relapsing-remitting course of human IBD, and are associated with dynamic changes in the gut microbiota and mucosal T cell responses (Jacobs et al., 2017). It should be noted, however, that *C1GalT1* deletion disrupts core O-glycan biosynthesis at a more fundamental level than altered fucosylation alone, truncating essentially all core 1–derived mucin glycans; this model therefore illustrates the general requirement for intact mucin glycosylation rather than a fucosylation-specific defect. During the remission period, an increase in colonic FoxP3^+^RORγt^+^ regulatory T cells is observed in these mice, which is positively correlated with the S24-7 family of bacteria, while during the relapse period, the expansion of pro-inflammatory FoxP3^–^RORγt^+^ T cells is associated with members of the *Clostridiales* order (Jacobs et al., 2017). This mechanism has clinical relevance—in patients with UC, exposure of the Tn antigen due to *Cosmc* gene mutations is similar, and inducible gene deletion in adult mice confirms that mucus barrier disruption precedes inflammation (Bergstrom and Xia, 2013). Additionally, polymorphisms in the *C1GALT1*/*Cosmc* adjacent genes in patients with Crohn’s disease are associated with dysbiosis (Jacobs et al., 2017). Defects in core 3 synthesis (such as the downregulation of C3GnT in the colon of UC patients leading to truncated mucin glycans) also weaken the barrier’s resistance to proteolytic degradation (Wang et al., 2022; Okumura and Takeda, 2024), while the upregulation of GnT-V in IBD, which increases β1,6-GlcNAc branching, promotes pathogen adhesion and inflammatory infiltration (Wang et al., 2022). The absence of sialylation modification (such as ST6GALNAC1) also increases mucus susceptibility (Okumura and Takeda, 2024).

Recent studies have found through non-invasive analysis of human fecal MUC2 that the O-glycan profile of mucus in IBD patients is significantly altered, affecting the metabolic functions of commensal bacteria (Fancy et al., 2024); *Fut2* gene knockout mice, due to impaired mucus barrier, show reduced microbiota diversity, and their dysbiotic microbiota produce pro-inflammatory metabolite lysophosphatidylcholine (LPC), which directly damages the epithelium and amplifies inflammation, a mechanism verified by fecal microbiota transplantation experiments (Tang et al., 2021). Notably, chronic inflammation can regulate *MUC2* expression through pathways such as NF-κB, forming a vicious cycle (Cheng et al., 2025). Therapeutic strategies to enhance intestinal fucosylation have shown protective effects in animal models (Zhong et al., 2025), highlighting the central role of this modification in maintaining barrier homeostasis.

Intestinal fucosylation is linked to systemic inflammation, including rheumatoid arthritis (RA), underscoring the “gut-joint axis” (Pan et al., 2024). In collagen-induced arthritis (CIA) mice, joint inflammation coincides with intestinal damage and reduced fucosylation—indicating that systemic inflammation disrupts intestinal fucosylation homeostasis. Oral undenatured type II collagen therapy reverses this defect: it restores fucosylation, repairs gut lesions, rebalances IL-17/IL-22 responses, and enriches beneficial bacteria (e.g., *Akkermansia, Bifidobacterium*), thereby recovering microbial α-diversity. These findings show that restoring oral tolerance—and with it, intestinal fucosylation—can ameliorate distant organ inflammation, revealing a “gut-target organ axis” as a therapeutic paradigm for systemic autoimmunity (Pan et al., 2024).

### 4.3 Metabolic diseases

The role of intestinal fucosylation in metabolic diseases such as obesity, non-alcoholic steatohepatitis (NASH), and ALD is remarkably complex and context-dependent. Evidence from mouse models has yielded seemingly contradictory findings, suggesting that the impact of fucosylation depends on the specific metabolic challenge. Adding to this complexity, in the context of acute systemic inflammation (e.g., from LPS exposure), Fut2-mediated fucosylation is protective, promoting faster weight recovery in a microbiota-dependent manner (Pickard et al., 2014). This suggests that the metabolic impact of fucosylation is highly dependent on the physiological state of the host—whether it is facing a chronic high-fat diet challenge or an acute inflammatory stress.

Under a high-fat Western diet, endogenous Fut2-mediated α1,2-fucosylation is harmful. Wild-type mice show suppressed intestinal *Fut2* expression and reduced α1,2-fucosylation—likely an adaptive protective response. *Fut2*-deficient mice resist obesity, glucose intolerance, and steatohepatitis despite increased caloric intake. This protection is entirely microbiota-dependent: it transfers to wild-type mice via co-housing and is abolished by antibiotics. Mechanistically, it results from microbiota-driven bile acid remodeling—increased fecal secondary bile acid excretion activates intestinal FXR signaling, which suppresses hepatic bile acid synthesis and enhances energy expenditure (elevated core temperature) and thermogenesis (Ucp1 upregulation in brown adipose tissue). Conversely, supplementing wild-type mice with α1,2-fucosylated glycans worsens metabolic dysfunction (Zhou R. et al., 2021). In contrast, the exogenous HMO 2’-FL protects against high-fat diet–induced obesity and glucose intolerance, associated with increased GLP-1 and PYY secretion, via modulation of the intestinal mucus layer (thicker sterile inner layer, upregulated *Muc2*, *Muc4*, and Muc13, and altered glycosyltransferase activity), enrichment of *Akkermansia* and *Bacteroides*, shifts in the fecal proteome, and regulation of the intestinal endocannabinoid system (Paone et al., 2024). Thus, the metabolic effects of fucosylated glycans depend on their source (endogenous vs. exogenous), structure, and physiological context.

Building on evidence that fucosylated prebiotics enrich *Akkermansia*, recent work tested whether *A. muciniphila* supplementation directly remodels the mucus layer in diet-induced obesity. Live *A. muciniphila* administration to high-fat diet–fed mice reduced weight gain and fat mass—and, crucially, altered host glycosylation (Paone et al., 2026). Though mucus thickness was unchanged, composition shifted markedly: cecal *Fut1* expression increased, while jejunal *Fut8* upregulation (induced by HFD) was reversed. Mass spectrometry confirmed these transcriptional changes translated into a modified mucin glycan profile—specific HFD-induced fucosylated structures disappeared after probiotic treatment. Thus, *A. muciniphila*—a mucin-specialist commensal—directly tunes host fucosylation to reshape the mucosal environment and counteract metabolic disease, independent of broad microbiota restructuring.

In alcohol-related liver disease (ALD), the role of fucosylation is reversed: duodenal biopsies from patients with alcohol use disorder show downregulation of α1,2-fucosylation; the expression of FUT2 protein in colonic epithelium and overall fucosylation levels are also significantly reduced (Zhou et al., 2020; Chen L. et al., 2025). Correspondingly, mice with intestinal epithelial cell-specific *Fut2* knockout (*Fut2*^Δ^ ^IEC^) are more sensitive to ethanol-induced liver injury, steatosis, and inflammation—this phenotype is not due to changes in ethanol metabolism but is microbiota-dependent: cohabitation with wild-type mice can completely protect *Fut2*^Δ^ ^IEC^ mice, and supplementation with α1,2-fucosylated HMO 2’-FL can also significantly improve liver pathology (Zhou et al., 2020; Chen L. et al., 2025). The protective mechanism operates through a dual pathway: (1) Inhibiting pathogen colonization—limiting the expansion of *Enterococcus faecalis*, which produces cytolysin and has been shown to drive the progression of ALD, in the intestine; (2) Maintaining liver NAD^+^ homeostasis—*Fut2* deficiency leads to a reduction in commensal bacteria carrying nicotinamidase (PncA), weakening the conversion of nicotinamide (NAM) to nicotinic acid (NA), and thereby blocking the *de novo* synthesis of NAD^+^ mediated by the Preiss–Handler pathway in the liver, exacerbating oxidative stress. This gut-liver axis causal chain has been confirmed by functional rescue experiments: colonizing alcohol-fed *Fut2*^Δ^ ^IEC^ mice with *E. coli* overexpressing *pncA* can restore NAD^+^ levels in the liver and significantly alleviate liver injury (Chen L. et al., 2025).

### 4.4 Colorectal cancer (CRC)

Aberrant mucin glycosylation is a well-established hallmark of CRC, driving tumorigenesis through dual mechanisms: erosion of the protective mucus barrier and induction of chronic inflammation. Beyond fucosylation defects, impaired terminal glycan extension leads to accumulation of truncated O-glycans—including the tumor-associated Tn and sialyl-Tn (sTn) antigens—which correlate with heightened mucosal inflammation and accelerated neoplastic progression, especially in patients with long-standing UC (Li Y. et al., 2025). Moreover, CRC epithelial cells display elevated levels of highly branched N-glycans that directly enhance cell adhesion and migration, thereby facilitating tumor invasion and metastasis. Critically, this glycosylation dysregulation extends beyond the local tumor microenvironment: aberrantly glycosylated IgA is consistently detected in the serum of CRC patients, establishing it as a functionally relevant and clinically accessible biomarker (Ding et al., 2022). Disruption of core O-glycan synthesis—exemplified by loss of the core 3 synthase B3GNT6—increases susceptibility to colitis and accelerates subsequent colorectal tumorigenesis in preclinical models, directly attributable to mucus barrier weakening (Okumura and Takeda, 2024; Li Y. et al., 2025).

Beyond the fucosylation defects detailed above, aberrant CRC glycosylation is concurrently characterized by hypersialylation—the excessive addition of sialic acid residues mediated by upregulated sialyltransferases (e.g., ST3GAL3/6). This generates a synergistic “sialylated-fucosylated” phenotype: fucosylated Lewis antigens (Le^x^/Le^y^) serve as scaffolds for subsequent sialylation, producing sialyl-Lewis antigens (sLe^x^/^a^) that engage E-selectin to drive tumor cell extravasation and metastasis (Nakagoe et al., 2000; Pinho and Reis, 2015). Notably, fucosylation and sialylation compete for the shared precursor UDP-GlcNAc; metabolic rewiring that favors sialic acid biosynthesis can thus indirectly suppress fucosylation in subsets of CRC cells, contributing to the glycan heterogeneity observed across tumors.

In CRC, disruption of the GDP-fucose synthesis pathway represents a core pathogenic mechanism—supported by convergent evidence across clinical genomics, preclinical modeling, molecular signaling, and therapeutic intervention. Clinically, loss-of-function alterations in *GMDS*—the gene encoding the rate-limiting enzyme in *de novo* GDP-fucose biosynthesis—are detected in 6–13% of CRCs, with marked enrichment in right-sided tumors (ascending and transverse colon). This genetic lesion directly impairs mucin fucosylation and is strongly associated with tumor initiation. Preclinically, systemic fucosylation-deficient mice (Fx-/-) accurately recapitulate this defect and develop spontaneous chronic colitis, sessile serrated lesions, and progression to adenocarcinoma in ∼40% of animals—predominantly in the cecum and right colon—preceded by epithelial barrier breakdown, microbiota dysbiosis, and sustained immune activation, establishing fucosylation deficiency as a primary driver of inflammation-associated carcinogenesis. Mechanistically, fucosylation is indispensable for Notch receptor activation; its absence abrogates *HES1* expression, resulting in disorganized crypt proliferation and impaired differentiation—a phenotype mirrored in human right-sided CRC and sessile serrated adenomas (SSAs), the principal precursors of these tumors (Wang et al., 2017). Critically, MUC2—the dominant intestinal gel-forming mucin—relies on fucosylation for structural integrity and immunoregulatory function; its under-fucosylation compromises barrier competence, elevates paracellular permeability, and synergizes with dysbiosis to fuel chronic inflammation and neoplastic transformation, aligning with the established paradigm that defective mucin glycosylation is a linchpin of gut barrier failure in CRC (Li Y. et al., 2025). Most compellingly, dietary fucose supplementation fully prevents—and reverses—disease progression in Fx-/- mice, restoring mucosal fucosylation via the salvage pathway and rescuing barrier function, immune homeostasis, and epithelial architecture (Wang et al., 2017). Together, these findings position the GDP-fucose pathway not merely as a biomarker but as a therapeutically targetable node at the intersection of host glycosylation, microbial ecology, and intestinal carcinogenesis.

### 4.5 Autism spectrum disorder

Genetic defects in glycosylation pathways exert systemic effects that extend beyond gastrointestinal malignancy and inflammation into neurodevelopmental disorders. In children with Autism Spectrum Disorder (ASD), genome-wide analyses reveal significant enrichment of single-nucleotide variants (SNVs) in glycosylation-related genes—including mucins (e.g., MUC4) and glycosyltransferases (e.g., B3GALNT2) (Liu et al., 2021). These variants are robustly associated with gut microbiome dysbiosis, supporting a causal model wherein host glycosylation deficiencies drive ASD pathogenesis by remodeling the intestinal microbial ecosystem toward a pro-inflammatory, dysbiotic state.

## 5 Therapeutic perspectives and future directions

### 5.1 Fucosylated prebiotics

**FIGURE 10:**
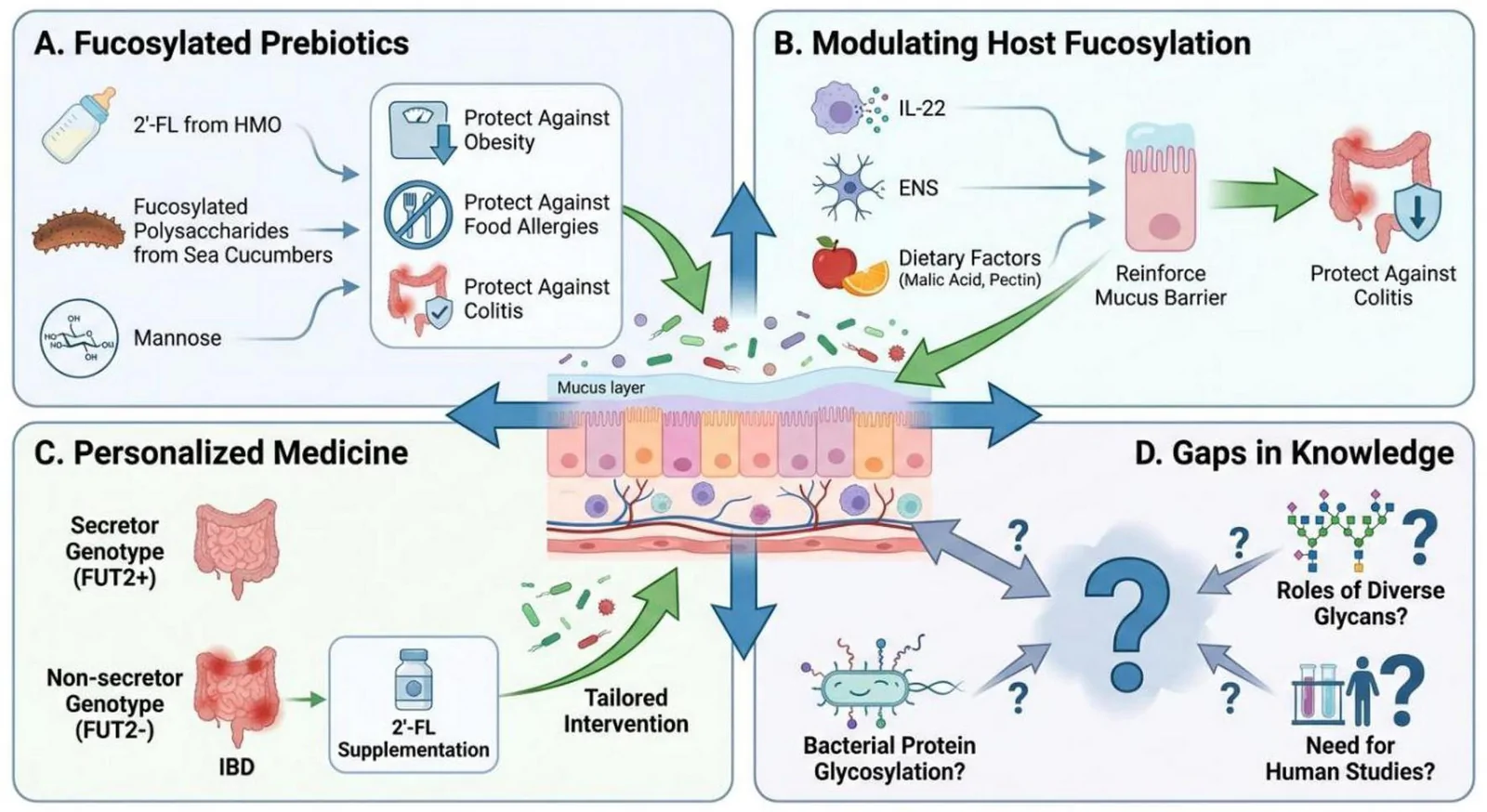
Fucosylation at the host–microbiota interface in gut health. Schematic overview of dietary and host fucosylation pathways and their therapeutic potential. **(A)** Fucosylated prebiotics. Fucose-containing glycans—human milk 2’-fucosyllactose (2’-FL), fucosylated polysaccharides from sea cucumbers, and mannose—protect against obesity, food allergies, and colitis. **(B)** Modulating host fucosylation. Epithelial fucosylation is regulated by IL-22, the ENS, and dietary factors (malic acid, pectin), and reinforces the mucus barrier to protect against colitis. **(C)** Personalized medicine. Non-secretor individuals (*FUT2*-) lacking terminal fucose on mucosal glycans show increased IBD susceptibility and may benefit from tailored 2’-FL supplementation. **(D)** Gaps in knowledge. Open questions include the roles of diverse glycans, bacterial protein glycosylation, and the need for human clinical studies. The central panel depicts the mucus layer separating the gut microbiota from the intestinal epithelium, positioning fucosylation as a bidirectional hub linking diet, microbes, and host physiology.

Fucosylated glycans can be used as prebiotics to restore gut homeostasis (Figure 10 and Table 6). The HMO 2’-FL protects against high-fat diet-induced obesity and glucose intolerance by reshaping the gut microbiota (Paone et al., 2024) and attenuates food allergy symptoms in mice (Chen X. et al., 2025). This therapeutic potential, together with the high abundance of 2’-FL in human milk and its near-absence from bovine milk—the basis of most infant formulas—has driven considerable interest in supplementing infant formula with 2’-FL (Walsh et al., 2020). Large-scale biotechnological production of 2’-FL is now feasible through enzymatic synthesis and engineered microbes (Zhou W. et al., 2021; Moya-Gonzálvez et al., 2024). Strategies include engineering gut microbial α-L-fucosidases for efficient synthesis of 2’-FL and 3’-FL (Moya-Gonzálvez et al., 2024) and using prophage-encoded FUTs, whose products can alleviate colitis in mice (Gao et al., 2025). These interventions are relevant for infant nutrition, potentially mitigating dysbiosis from cesarean birth (Tonon et al., 2021). Other fucosylated polysaccharides from sea cucumbers (Li S. et al., 2024) and sialylated/fucosylated glycans from goat milk (Li Y. et al., 2025) also show therapeutic potential against obesity and food allergies, respectively.

**TABLE 6:** Summary of therapeutic strategies targeting intestinal fucosylation.

Name	Content/function	Citation (PMID)	Study model
2’-FL	Protects against high-fat diet-induced obesity and glucose intolerance by reshaping gut microbiota.	(Paone et al., 2024)	Mouse
2’-FL	Attenuates food allergy symptoms in mice.	(Chen X. et al., 2025)	Mouse
Engineered microbial α-L-fucosidases	Enable efficient synthesis of fucosylated prebiotics like 2’-FL and 3’-FL.	(Moya-Gonzálvez et al., 2024)	*In vitro* (enzymatic synthesis)
Prophage-encoded FUTs	Products can alleviate colitis in mice.	(Gao et al., 2025)	Mouse
Fucosylated polysaccharides (from sea cucumber)	Show therapeutic potential against obesity.	(Li S. et al., 2024)	Mouse
Sialylated/fucosylated glycans (from goat milk)	Show therapeutic potential against food allergies.	(Li Y. et al., 2025)	Mouse
Mannose	Prevents high-fat diet-induced obesity, adiposity, and liver steatosis in mice.	(Sharma et al., 2018)	Mouse
Kiwifruit polysaccharides	Enhance IL-22 to alleviate UC.	(Li S. et al., 2024)	Mouse
IL-22	Critical for host protection against rotavirus infection.	(Schnepf et al., 2021)	Mouse
N-acetylspermidine (from *A. muciniphila*)	Enhances host fucosylation and protects against colitis.	(Yao et al., 2025)	Mouse
Malic acid (from *Pithecellobium clypearia*)	Enhances fucosylation to protect against colitis.	(Zhong et al., 2025)	Mouse
Pectin/ inulin (dietary fibers)	Increase fucosylated mucin O-glycans.	(Zhao et al., 2023; Zhao et al., 2025)	Mouse (Zhao et al., 2023); *in vitro* (fecal fermentation, HT29-MTX cells) (Zhao et al., 2025)
Transgenic mucin crosslinkers	Increase ileal fucosylation and reduce susceptibility to colitis and infection.	(Gouyer et al., 2015)	Mouse (transgenic)

2’-FL, 2’-fucosyllactose; 3’-FL, 3’-fucosyllactose; FUT, fucosyltransferase; UC, ulcerative colitis; IL-22, interleukin-22; PMID, PubMed identifier.

Beyond fucosylated structures, simple monosaccharides like mannose can prevent high-fat diet-induced obesity, adiposity, and liver steatosis in mice by increasing the Bacteroidetes-to-Firmicutes ratio and reducing dietary energy harvest (Sharma et al., 2018). This protective effect is specific to mannose (not galactose) and is only effective when administered early in life, highlighting a critical developmental window for intervention (Sharma et al., 2018).

### 5.2 Modulating host fucosylation

Targeting the host’s endogenous fucosylation machinery offers a nuanced therapeutic strategy. Key regulators include IL-22, which can be enhanced by kiwifruit polysaccharides to alleviate UC in mice (Li S. et al., 2024) and is critical for protection against rotavirus in mice (Schnepf et al., 2021). The ENS also shapes epithelial α1,2-fucosylation via VIP-expressing neurons in mice (Lei et al., 2022). Microbial metabolites, such as N-acetylspermidine from *A. muciniphila*, enhance fucosylation and protect against colitis in mice (Yao et al., 2025). Dietary interventions are also effective in mouse models: malic acid from *Pithecellobium clypearia* enhances fucosylation to protect against colitis (Zhong et al., 2025), while specific fibers like pectin or inulin increase fucosylated mucin O-glycans in mice (Zhao et al., 2023) and in *in vitro* human fecal fermentation and HT29-MTX cell systems (Zhao et al., 2025). Conversely, low-fiber diets suppress epithelial α1,2-fucosylation in mice (Shiratori et al., 2024). Additionally, physically reinforcing the mucus barrier with transgenic mucin crosslinkers increases ileal fucosylation, enriches for *Lactobacillus*, and reduces susceptibility to colitis and infection in transgenic mice (Gouyer et al., 2015).

### 5.3 Personalized medicine

Inter-individual variability in fucosylation, governed by *FUT2* genetics, necessitates a personalized medicine approach. The “non-secretor” phenotype, resulting from loss-of-function *FUT2* mutations, is associated with altered IBD risk and a distinct gut microbiome (Cheng et al., 2021; Sharma et al., 2025). In early life, the infant’s own *FUT2* genotype is the dominant factor shaping the microbiome (Thorman et al., 2023). Furthermore, probiotic efficacy depends on host-bacterial genetic interplay (Geng et al., 2025). Therefore, *FUT2* genotyping could guide personalized interventions, such as 2’-FL supplementation for non-secretor IBD patients.

### 5.4 Experimental methodologies for intestinal fucosylation

Dissecting host fucosylation and fucose-utilizing microbiota requires orthogonal technical pipelines. For host glycans, LC-MS/MS glycomics resolves site-specific fucosylation of mucins/glycolipids (Nilsson et al., 2024), glycoproteomics identifies fucosylated carrier proteins, UEA-I lectin-based assays detect α1,2-fucosylation, and confocal microscopy maps spatial distribution of fucosylated epitopes along the gastrointestinal axis. For microbial communities, metagenomics profiles fucosidase gene repertoires, metatranscriptomics captures real-time activation of fucose utilization pathways *in vivo*, and ^13^C-fucose stable isotope probing tracks active fucose consumers (El Kaoutari et al., 2013). Current gaps include limited coverage of labile mucin O-glycans and poor linkage between specific glycan structures and cognate microbial consumers—limitations being addressed by single-cell glycomics and high-resolution meta-oligotyping.

### 5.5 Gaps in knowledge and future research

Future research must address several knowledge gaps. A key unresolved question is how to disentangle fucosylation effects from other intestinal glycosylation pathways *in vivo*, given their overlapping regulatory networks and synergistic mucosal defense functions. Current tools also lack specificity to independently perturb α1, 2-, α1, 3-, and α1,6-fucosylation, limiting mechanistic dissection of linkage-specific functions. Further advances in site-specific glycan sequencing are needed to resolve co-occurring modifications on individual glycan chains and define their independent contributions to disease.

The specific biological roles of diverse fucosylated glycans beyond 2’-FL, such as 3-fucosyllactose or di-fucosylated O-glycans, remain largely unknown (Zhao et al., 2023; Moya-Gonzálvez et al., 2024). A parallel frontier is targeting bacterial protein glycosylation systems, which mediate host-pathogen interactions and immune evasion (Zhu and Wu, 2016), representing a novel anti-infective strategy.

Understanding the interplay between host regulatory pathways—such as IL-22 signaling (Li S. et al., 2024), ENS activity (Lei et al., 2022), and microbial metabolites (Yao et al., 2025)—is crucial for designing combination therapies.

Validating animal model findings in human studies is a high priority. Research should also investigate systemic effects, like IgG afucosylation in aging (Zhang and Li, 2026), and the mechanisms by which microbes regulate host mucin glycosylation (de Ram et al., 2024). Progress requires novel tools, such as activity-based probes for microbial fucosidases (Luijkx et al., 2021).

Finally, it is critical to characterize how novel dietary components impact the glycome; for instance, yeast-synthesized whey protein has a different N-glycan profile than bovine whey, which differentially alters human gut microbial communities, showing that glycosylation, not just protein sequence, dictates biological impact (Bolino et al., 2025).

## 6 Conclusion

Intestinal fucosylation is a dynamic, host–microbe–regulated process. Host genetics—especially *FUT2* status—set baseline mucosal glycan patterns, which immune signals (e.g., IL-22), maternal immunity, and neuropeptides fine-tune. Crucially, the microbiota drives fucosylation via a feedback loop: microbes induce host FUTs, and host fucose shapes microbial composition—even under stress like radiation.

Fucosylated glycans on gut surfaces or in breast milk serve as selective nutrients for commensals. In infancy, fucosylated HMOs and glycoproteins enrich *Bifidobacterium* and suppress pathobionts—dependent on bacterial α-L-fucosidases. This drives cross-feeding and syntrophy, making host fucose presentation an early-life ecological filter that guides microbial colonization, barrier defense, and immune development.

Disruption of this axis impairs barrier function and mutualism, increasing risk for colitis, IBD, obesity, and metabolic syndrome. Gut dysbiosis and leakiness also fuel systemic disease—via gut–joint and gut–brain axes—linking early-life fucosylation to lifelong health.

Therapeutically, natural fucosylated prebiotics (e.g., HMOs, fucoidan, sea cucumber glycoproteins) selectively boost beneficial symbionts and improve metabolic health in preclinical models. Enzymatically synthesized fucosylated oligosaccharides offer precise, structure-defined alternatives. Together, these strategies aim to restore host–microbe homeostasis for precision treatment of inflammatory, metabolic, and neurodevelopmental disorders.
